# Intrusive Memories and Voluntary Memory of a Trauma Film: Differential Effects of a Cognitive Interference Task After Encoding

**DOI:** 10.1037/xge0000598

**Published:** 2019-04-25

**Authors:** Alex Lau-Zhu, Richard N. Henson, Emily A. Holmes

**Affiliations:** Medical Research Council Cognition and Brain Sciences Unit, School of Clinical Medicine, University of Cambridge, United Kingdom; Medical Research Council Cognition and Brain Sciences Unit, School of Clinical Medicine, University of Cambridge, United Kingdom; Medical Research Council Cognition and Brain Sciences Unit, School of Clinical Medicine, University of Cambridge, United Kingdom

**Keywords:** consolidation, intrusive memories, involuntary memory, mental imagery, posttraumatic stress disorder

## Abstract

Methods to reduce intrusive memories (e.g., of traumatic events) should ideally spare voluntary memory for the same event (e.g., to report on the event in court). Single-trace memory accounts assume that interfering with a trace should impact both its involuntary and voluntary expressions, whereas separate-trace accounts assume these two can dissociate, allowing for *selective* interference. This possibility was investigated in 3 experiments. Nonclinical participants viewed a trauma film followed by an interference task (Tetris game-play after reminder cues). Next, memory for the film was assessed with various measures. The interference task reduced the number of intrusive memories (diary-based, Experiments 1 and 2), but spared performance on well-matched measures of voluntary retrieval—free recall (Experiment 1) and recognition (Experiments 1 and 2)—challenging single-trace accounts. The interference task did not affect other measures of involuntary retrieval—perceptual priming (Experiment 1) or attentional bias (Experiment 2). However, the interference task did reduce the number of intrusive memories in a laboratory-based vigilance-intrusion task (Experiments 2 and 3), irrespective of concurrent working memory load during intrusion retrieval (Experiment 3). Collectively, results reveal a robust dissociation between intrusive and voluntary memories, having ruled out key methodological differences between how these two memory expressions are assessed, namely cue overlap (Experiment 1), attentional capture (Experiment 2), and retrieval load (Experiment 3). We argue that the inability of these retrieval factors to explain the selective interference is more compatible with separate-trace than single-trace accounts. Further theoretical developments are needed to account for this clinically important distinction between intrusive memories and their voluntary counterpart.

We are grateful to Brian Levine, who shared the protocol of the Autobiographical Interview for the recall task in Experiment 1; Elze Landkroon, who provided inter-rater ratings of the recall task; Colin MacLeod and Ben Grafton, who advised on the dot-probe task in Experiment 2; Joni Holmes, who advised on the vigilance-intrusion task design in Experiment 3; David Hayes who built the tapping keyboard in Experiment 3; Peter Watson, who provided statistical advice in Experiment 3; Ella James, who provided inter-rater ratings of the intrusion diaries in Experiments 1–3; and Lalitha Iyadurai, who provided assistance in coding diary cues in Experiments 1 and 2. We are also grateful for the British Film Institute National Archives, LyleBailie International, Kino International, and David Large at the Royal College of Surgeons of Edinburgh for use of film clips as part of our materials. Alex Lau-Zhu was supported by a Cambridge International Scholarship awarded by The Cambridge Commonwealth, European and International Trust. Richard N. Henson is supported by the United Kingdom Medical Research Council (MRC) Intramural Programme (SUAG/010 RG91365). Emily A. Holmes was supported by the United Kingdom MRC Intramural Programme (MC-A060-5PR50). Funding to pay the Open Access publication charges for this article is provided by the MRC.

Intrusive memories of a traumatic event, or more simply *intrusions*, comprise the core clinical feature of acute stress disorder (ASD) and posttraumatic stress disorder (PTSD; *Diagnostic and Statistical Manual of Mental Disorders*, fifth ed. [*DSM–5*]; American Psychiatric Association [APA], 2013). For example, after a road traffic accident, one may experience intrusive visual images of a red car zooming toward oneself, accompanied by disabling fear. The intrusive nature of these emotional memories entails them springing to mind *involuntarily* (APA, 2013), that is, popping to awareness unbidden. In contrast, voluntary retrieval of a trauma involves deliberate attempts to remember the event (Berntsen, 2009; Conway & Pleydell-Pearce, 2000). Established evidence-based clinical interventions for PTSD, such as trauma-focused cognitive–behavioral therapy (National Collaborating Centre for Mental Health, 2005), help to reduce the occurrence of *intrusive* memories of trauma; however, they do not seek to erase all memories of the trauma (Holmes, Sandberg, & Iyadurai, 2010). That is, psychological treatments should ideally preserve voluntary access to recollections of the trauma so that the patient can discuss their trauma when required. For example, a trauma victim may be asked to report on the event for legal reasons; a journalist may need to conjure up details of traumatic events to pitch a news story; a firefighter may wish to reflect on a trauma for future safety even if they may not wish the event to intrude. Thus, the impacts of successful therapy are selective—they may alter some aspects of memory but not others.

Experimental psychopathology findings suggest that the impact of a cognitive intervention on different types of memory of an emotional episode can indeed be selective: the occurrence of intrusive memories can be altered while leaving voluntary memory seemingly intact. A series of experiments have shown that, *after* viewing a trauma film, engaging in certain interference tasks (e.g., performing a cognitive task such as Tetris game-play after a film reminder cue) reduces the number of intrusive memories of the film (diary-based measure), but has no detectable effect on voluntary memory of the same film (as indexed in all of the following studies by spared performance on recognition memory: Deeprose, Zhang, Dejong, Dalgleish, & Holmes, 2012; Holmes, James, Coode-Bate, & Deeprose, 2009; Holmes, James, Kilford, & Deeprose, 2010; James et al., 2015). This *selective interference effect* on intrusive (involuntary) memory–but not voluntary memory–has been shown across at least 11 experiments using trauma films (Bourne, Frasquilho, Roth, & Holmes, 2010: Experiment 1; Brewin & Saunders, 2001; Deeprose et al., 2012: Experiment 2; Holmes, Brewin, & Hennessy, 2004: Experiments 1–3; Holmes et al., 2009; Holmes, James, et al., 2010: Experiments 1 and 2; James et al., 2015; Krans, Näring, Holmes, & Becker, 2010). Interestingly, intrusive and voluntary memory of a trauma film can also be differentially modulated by other psychological (Hagenaars & Arntz, 2012; Jobson & Dalgleish, 2014; Krans, Näring, & Becker, 2009; Krans, Näring, Holmes, & Becker, 2009; Pearson, Ross, & Webster, 2012) and pharmacological procedures (Bisby, Brewin, Leitz, & Valerie Curran, 2009; Das et al., 2016; Hawkins & Cougle, 2013).

Further experiments have sought to determine the boundary conditions of the interference effects on intrusive memories. Cognitive interference tasks that are visuospatial (e.g., complex finger tapping or the computer game Tetris) are claimed to be more effective than verbal tasks (e.g., counting backward or the computer game Pub Quiz) in reducing intrusion rates (see Brewin, 2014, for a review), although there are some exceptions (e.g., Hagenaars, Holmes, Klaassen, & Elzinga, 2017; Krans, Langner, Reinecke, & Pearson, 2013). A modality-specific hypothesis has been proposed, which postulates that sufficiently demanding visuospatial (but not verbal) tasks would preferentially disrupt the visual imagery that underlines later visual-based intrusions (Brewin, 2014; Holmes et al., 2004; Holmes, James, et al., 2010). Nevertheless, an alternative line of enquiry suggests that the important factor is general working memory (WM) load and not modality, which deserves further exploration (Engelhard, Van Uijen, & Van den Hout, 2010; Gunter & Bodner, 2008; van den Hout & Engelhard, 2012). In this paper, however, we will restrict ourselves to a visuospatial task—the computer game Tetris (Lau-Zhu, Holmes, Butterfield, & Holmes, 2017)—which has been used successfully in many of the aforementioned studies in generating the interference effect.

The interference effect on subsequent intrusions of the film occurs when the cognitive task is performed both *during* (Bourne et al., 2010; Holmes et al., 2004; Krans et al., 2010) and *after* the trauma film, including minutes to hours after (Deeprose et al., 2012; Holmes et al., 2009; Holmes, James, et al., 2010), and even one to four days after (Hagenaars et al., 2017; James et al., 2015). In the latter case at longer time intervals, the interference effect is conditional on the cognitive task being preceded by a reminder cue, which is presumably needed to reactivate the memory trace such that it is labile and can be disrupted (Visser, Lau-Zhu, Henson, & Holmes, 2018). The necessity of the reminder cue at shorter time intervals (after the film) is unclear, though it has typically been included in the aforementioned studies. Beyond films with traumatic content, intrusive memories can also be induced by films with overly positive (Davies, Malik, Pictet, Blackwell, & Holmes, 2012) or depression-linked material (Lang, Moulds, & Holmes, 2009). Such intrusions can be modulated by interference procedures too (Davies et al., 2012), suggesting that the mechanisms apply to emotional memories more broadly. Nonetheless, a pivotal issue remains unresolved from the last two decades of trauma film research: how can such interference tasks selectively reduce the number of intrusions while leaving voluntary memory intact?

The distinction between intrusive (involuntary) memories and their voluntary counterparts is intriguing, because it is rarely considered by conventional memory theories. A widely agreed dichotomy is between declarative versus nondeclarative memory *systems* (Squire, 1992; Squire & Zola, 1996), with declarative memory often subdivided into episodic versus semantic memory (Tulving, 1972, 2002). Consistent with this declarative/non-declarative dichotomy, existing research on emotional memory has shown that nondeclarative memory, for example, the startle response to fear-eliciting stimuli, can be modulated by a pharmacological manipulation while leaving declarative memories intact, as indexed for instance by self-reported fear or learnt contingencies for receiving a shock (Kindt, Soeter, & Vervliet, 2009; Soeter & Kindt, 2010, 2012, 2015; for a recent review see Visser et al., 2018). Yet because both intrusive and voluntary memories of traumatic material entail retrieval of verbalizable information about the same episode, both would normally be associated with a declarative/episodic memory system (Berntsen, 2009; Rubin, Boals, & Berntsen, 2008; Tulving, 1972, 2002). We call such accounts *single-trace* theories.

Note that another common dichotomy is between explicit versus implicit memory (Schacter, 1987, 1992), which refers to differences in *awareness*—the phenomenological experience of retrieving a memory (regardless of intention). Because intrusions and voluntary retrievals are both experienced consciously, both would also normally be considered examples of explicit memory.

An alternative class of theories assumes that intrusions and voluntary memories arise from different memory systems (Bisby & Burgess, 2017; Brewin, 2014; Brewin, Dalgleish, & Joseph, 1996; Brewin, Gregory, Lipton, & Burgess, 2010; Jacobs & Nadel, 1998), some of which were inspired by other theories proposing independent systems for processing of imagery-based and non–imagery-based information (e.g., Brown & Kulik, 1977; Johnson & Multhaup, 1992; Paivio, 1971). We call these *separate-trace* theories.

Below, we first expand on key single-trace and separate-trace accounts and their predictions regarding selective interference effects. We then elaborate on key methodological (retrieval-based) differences that might have confounded prior comparisons of intrusions versus voluntary retrieval. Finally, we introduce how the present series of experiments address these methodological issues, and therefore inform the theoretical debate about this clinically important interference effect.

## Discrepancy Between Intrusive (Involuntary) and Voluntary Memory: Theoretical Perspectives

### Single-Trace Theories

These theories are mostly drawn from the literature on episodic and autobiographical memories, with the underlying assumption that both involuntary and voluntary memories are derived from the same memory system, differing in how those memories are retrieved based only on differences in retrieval *intention* (Richardson-Klavehn & Bjork, 1988) or possibly retrieval *mode* (Tulving & Thomson, 1973). A prominent view, based on the standard consolidation theory (Squire & Zola-Morgan, 1991), posits that episodic/declarative memories are initially encoded in the hippocampus and then gradually consolidate into the neocortex over hours or days (McGaugh, 2000, 2004). This broad system-level view is largely silent on the distinction between intrusive and other forms of episodic memory, and thus would assume that interfering with an episodic trace (through postencoding interference) should impact both intrusive and voluntary memories.

The same assumption is echoed by key theories on autobiographical memory, which either propose a self-memory system (Conway & Pleydell-Pearce, 2000) with a specialized storage for rich sensory-perceptual details (Conway, 2001), or portray involuntary memory as a basic mode of remembering (Berntsen, 1996, 1998, 2009, 2010; Berntsen & Rubin, 2014; Rubin et al., 2008; Staugaard & Berntsen, 2014). Both theories agree that involuntary and voluntary memories operate on the same memory system, sharing encoding and consolidation processes, but differing only in retrieval mechanisms. Thus, these theories would also predict that interfering with an episodic trace (through postencoding interference) should impact both intrusive and voluntary memories.

### Separate-Trace Theories

Alternative perspectives raise the possibility that more than one memory trace underlie intrusive and voluntary memory. Such multirepresentational approaches are prevalent in the clinical literature on information-processing in PTSD (Dalgleish, 2004; for a review), and have a long tradition in cognitive psychology (e.g., Brown & Kulik, 1977; Johnson & Multhaup, 1992; Paivio, 1971).

One such influential account is dual representation theory (Brewin, 2014; Brewin et al., 1996), which proposes that two traces are formed at the time of trauma: verbally accessible memory (VAM) consisting of representations of the trauma that are integrated with the wider autobiographical memory system, and situationally accessible memory (SAM) consisting primarily of sensory and affective components that are not integrated in this system. More recent developments of the dual representation theory propose that intrusive memories are governed by a specialized, long-term perceptual memory system supporting autobiographical experiences, which can be only accessed automatically and is separate from the episodic memory system (Brewin, 2014). To support this, Brewin (2014) also draws on the notion that (conscious) reexperiencing symptoms in PTSD result partly from enhanced perceptual priming of trauma stimuli (Ehlers & Clark, 2000), which is a form of *implicit* (unconscious) memory arising from a nondeclarative memory system (Schacter, 1992). In terms of neural circuitry, intrusive memory representations are believed to result from associations between processing in the insula (internal representations of emotional states) and the dorsal visual stream (sensory representations), via the potentiated amygdala functioning after stress exposure alongside weakened hippocampal activity (Bisby & Burgess, 2017; Brewin et al., 2010). In sum, separate-trace accounts—such as dual representation theory—permit a dissociation between intrusive/involuntary (e.g., SAM; long-term perceptual representations linked to priming) and voluntary memories of trauma (e.g., VAM; ordinary episodic representations).

## Discrepancy Between Intrusive (Involuntary) and Voluntary Memory: Methodological Considerations

To explain an interference effect that is selective to intrusions, single-trace theories need to assume different *retrieval* processes underlying intrusions and voluntary memories. To demonstrate this, it is important to control for other differences in the way intrusions and voluntary memories are assessed, beyond the involuntary-voluntary dichotomy (the so-called retrieval intentionality criterion; Schacter, Bowers, & Booker, 1989). The previous trauma-film studies demonstrating selective interference have failed to consider the methodological differences that are inherent to most commonly used measures of intrusions (e.g., diaries) versus voluntary memory (e.g., recognition tasks). Thus, the main aim of the present study was to improve methodology by better matching the types of measures of memory, with the possibility that interference effects (putatively on consolidation of the memory trace) would then no longer dissociate involuntary from voluntary memory, supporting the hypothesis that interference affects the same underlying trace as assumed by single-trace accounts. However, if the selective interference on intrusions still occurs when controlling for differences in retrieval factors across measures, then separate-trace theories would seem more likely than single-trace theories.

**Figure 1:**
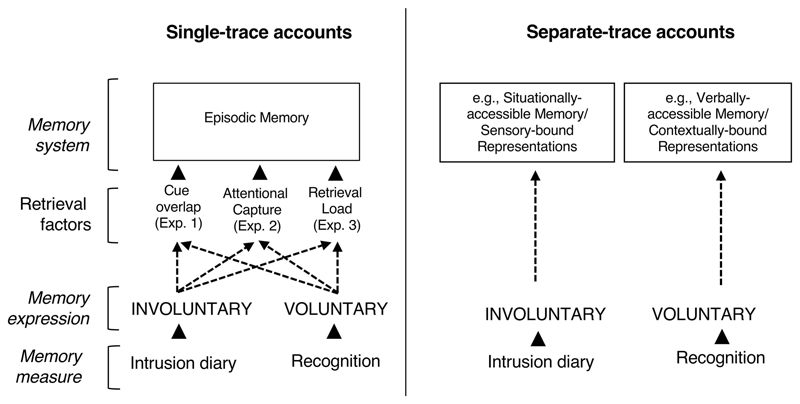
Schematic overview of single-trace versus separate-trace accounts of intrusive and voluntary memory. The relationships between memory measure, memory expression, and memory systems are fleshed out in the text for each type of account. Our series of experiments aimed to rule out three key retrieval factors informed by single-trace accounts in three experiments. Examples of separate-trace accounts based on Bisby and Burgess (2017); Brewin (2014); Brewin et al. (1996); Brewin et al. (2010).

Informed by foundational memory theories (Baddeley, Eysenck, & Anderson, 2009), as well as prominent accounts on involuntary autobiographical memory (Berntsen, 2009), we have identified differences between intrusion diaries and recognition tasks in three key aspects in the retrieval context or retrieval factors (see Figure 1), which could explain the selective interference (i.e., the apparent intrusion/recognition dissociation due to interference tasks found in trauma-film studies). Baddeley and colleagues (2009) presented seven textbook retrieval principles, three of which we considered in our study, namely retrieval mode (i.e., retrieval intention), cue-target strength (i.e., cue overlap), and attention to cues (i.e., a combination of attentional capture and retrieval load). These principles also broadly overlap with those considered important for involuntary memories as postulated by Berntsen (2009), namely retrieval intention, external cues, and attentional factors (cue saliency and diffuse attentional state). We expand on these below.

### Cue Overlap

This retrieval factor refers to the overlap between information presented at retrieval (e.g., retrieval cues) and information presented at encoding (Baddeley et al., 2009). It is established that the greater the retrieval-encoding overlap, the greater the chance of retrieving the full memory (Tulving & Thomson, 1973). A recognition task typically asks participants to distinguish old items that they encountered previously from new items that they did not. The *old* items can be *copy cues*, such as stills from the trauma film (James et al., 2015; James, Lau-Zhu, Tickle, Horsch, & Holmes, 2016). In contrast, copy cues are absent in the diary measure.

Some may argue that intrusions can be triggered by incidental cues in everyday life (Berntsen, 2009; Conway, 2001; Michael, Ehlers, Halligan, & Clark, 2005)—for example, when passing a red car in the street that resembles the one that was seen to crash in a trauma film—but these cues are unlikely to perfectly match visual elements of the original film like copy cues. The high cue-overlap in an experimental recognition task is arguably more effective at aiding access to visual memories than the low cue-overlap in everyday cues that prompt intrusions. If so, recognition tasks could be more robust to weakening of a memory trace, removing any effect of interference, and resulting in an interference effect that appears selective to the intrusion diary.

### Attentional Capture

This retrieval factor refers to the extent that initial exogenous attention is given to potential retrieval cues (Baddeley et al., 2009). Attention to relevant/salient sensory cues is considered to be a prominent retrieval route (Cabeza, Ciaramelli, Olson, & Moscovitch, 2008). The autobiographical memory literature also supports the notion that salient cues (e.g., attributable to motivational factors such as worries and everyday concerns) raise the probability of involuntary memories coming to mind (Berntsen, 2009).

In typical recognition tasks, attention is initially focused on the external retrieval cues as per instructions. In contrast, one could argue that in everyday life (e.g., diary measure), the initial focus of attention is rarely on potential cues; one is instead focusing on another task at hand. Such initially unattended cues, however, may subsequently capture attention, and then increase the likelihood of cue-elicited intrusions. The interference task may reduce intrusion likelihood by disrupting the extent of such attentional capture. Thus, it is at least conceivable that such a disruption of attentional capture is irrelevant to tasks in which attention is already oriented to cues (e.g., no attentional capture in recognition tasks, hence apparent spared performance), but is more apparent when cues are initially unattended (e.g., as assumed for the diary intrusions).

### Retrieval Load

This retrieval factor refers to the amount of cognitive resources available during retrieval to support the activation of the memory trace (Baddeley et al., 2009), including goal-directed retrieval (Cabeza et al., 2008; Conway & Pleydell-Pearce, 2000). The more resources available, the more these can be dedicated for memory activation. For example, resources in WM appear to be help form and maintain mental imagery (Baddeley & Andrade, 2000). Further, diffuse attentional states (e.g., low task demands leaving cognitive resources available) can promote involuntary recollections (Ball, 2007; Barzykowski & Niedźwieńska, 2018; Berntsen, 2009; Schlagman & Kvavilashvili, 2008; Vannucci, Pelagatti, Hanczakowski, Mazzoni, & Paccani, 2015).

One could argue that tasks assessing for recognition memory consume cognitive resources, especially if retrieval involves recollection (Yonelinas, 2002). In contrast, intrusive imagery-based memories might be more likely to be reported in the diary when relatively more WM resources are available (because task demands are low). Hence, variations in the strength of a memory trace might be more apparent in retrieval contexts that encourage (intrusive) memory activation in the first place (e.g., presumably in low retrieval-load in the diary), which in turn could more sensitive to reveal interference effects. In contrast, such variations might be less apparent in retrieval contexts that leave fewer resources for intrusive memory activation (e.g., presumably high retrieval-load in recognition tasks; possibly also in other involuntary-based tasks, e.g., see Experiment 2).

## Overview of Experiments

**Figure 2:**
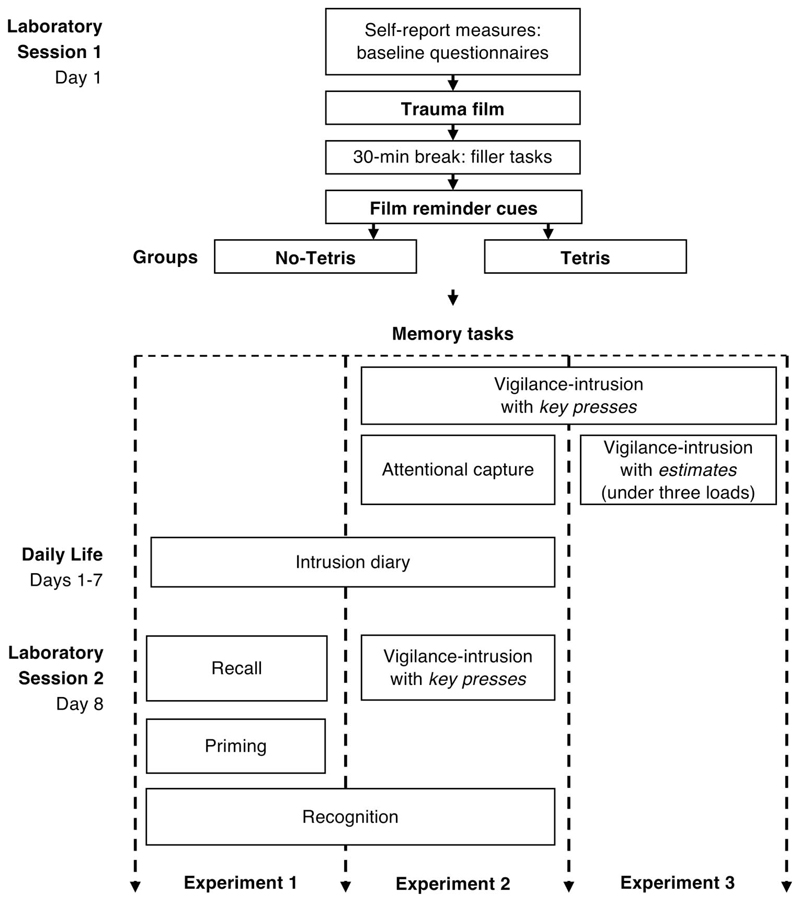
Schematic overview of the experimental procedures, highlighting the similarities and differences between memory measures across the current three experiments. Experiment 3 included an additional group that is not depicted (Tetris-only; without film reminder cues).

In the present series of experiments, we addressed the above three retrieval factors, which may have confounded previous comparisons of involuntary versus voluntary memory for traumatic film material. Figure 2 provides an overview of the procedure across experiments. In all experiments, participants watched a film with traumatic content, and then after a short delay, one group received film reminder cues followed by interference, that is, Tetris game-play (*reminder-plus-Tetris* group). The second (control) group received the film reminder cues but then sat quietly (*reminder-only* group). In line with previous studies (Deeprose et al., 2012; Holmes et al., 2009; Holmes, James, et al., 2010), we chose a 30-min delay between encoding and interference, as this is thought to fall within the time window of memory consolidation (up to 6 h postencoding; Nader, Schafe, & Le Doux, 2000), in which the memory is hypothesized to remain labile after encoding. Relevant to clinical translation, a 30-min delay is also considered reasonable time after an event to allow someone to be reached by postaccident and emergency interventions in the United Kingdom (National Audit Office, 2017) and the United States (Carr, Branas, Metlay, Sullivan, & Camargo, 2009).

Memory for the trauma film was then assessed by a battery of memory tasks, which were administered at two time points (see Figure 2): soon after the interference task within the same first session (Experiments 2 and 3) and/or a week later at follow-up (Experiments 1 and 2). The combination of these memory tasks was designed to address key methodological differences in retrieval factors (mainly cue overlap, attentional capture, and retrieval load) between the intrusion diary (measure of involuntary memory) and typical recognition memory tasks (measure of voluntary memory), as we explain in more detail later for each experiment.

Overall, we predicted fewer intrusions in the reminder-plus-Tetris group than the reminder-only group, but no difference between groups on recognition memory (Experiments 1 and 2). If some of the other new memory measures revealed an interference effect (in addition to the intrusion diary), then this would help isolate those retrieval factors that are important to allow for an apparent selective interference on intrusions (see Figure 1). For example, finding that an interference task *does* affect voluntary memory when there is low cue-overlap comparable to the intrusion diary (e.g., free-recall task in Experiment 1) would furthermore support single-trace accounts, which assume that the selectivity of interference arises at the time of retrieval (i.e., a matter of differential sensitivity to accessing the trace, which is removed once key retrieval factors are controlled for). Moreover, establishing that the size of the interference effect on intrusive/involuntary memory varies—depending on specific retrieval contexts—would also point toward retrieval factors that can produce an apparent selective interference on intrusions, assuming that measures of voluntary memory are unmatched to measures of intrusive/involuntary memory in such factors. If, however, an obvious retrieval factor cannot be identified that differentiates the memory measures (other than voluntary vs. involuntary), then the results would be more consistent with separate-trace theories, in which postencoding interference is allowed to affect one memory system but not the other.

## Experiment 1: Cue Overlap

The first aim of Experiment 1 was to replicate the pattern of selective interference on intrusive memory while sparing recognition memory (Deeprose et al., 2012; Holmes et al., 2009; Holmes, James, et al., 2010; James et al., 2015). The second aim was to test whether differences found between intrusions versus recognition genuinely reflected a distinction between involuntary versus voluntary retrieval (retrieval intention), rather than simply the effect of having higher cue-overlap in the recognition task (Tulving & Thomson, 1973) than in the diary. We tested this by factorially crossing retrieval intention with degree of cue overlap. This two-by-two factorial design was completed by adding two new memory measures of the film: free recall and perceptual priming (see the Method section for details). Whereas the diary can be considered as an *involuntary* measure with *low* cue-overlap, recognition memory can be considered as a *voluntary* measure with *high* cue-overlap; free recall can be considered example of a *voluntary* measure (like recognition) but with *low* cue-overlap (like the diary), while priming can be considered as example of an *involuntary* measure (like the diary) but with *high* cue-overlap (like recognition). Each participant completed all these four measures.

### Hypotheses

We predicted that the reminder-plus-Tetris group would have significantly fewer diary intrusions (summed across Days 1–7) compared with the reminder-only (control) group, but there would be no significant group differences on recognition performance (Day 8). If this were found, then two following alternative hypotheses were investigated. If the intrusion/recognition dissociation reflects methodological differences in cue overlap, then the reminder-plus-Tetris group (compared with the reminder-only group) would also show reduced voluntary memory in the context of low cue-overlap (lack of copy cues), that is, reduced performance on free recall. Alternatively, if the intrusion/recognition dissociation reflects a genuine distinction between involuntary and voluntary memory, then we predicted that the reminder-plus-Tetris group (compared with the reminder-only group) would also show reduced involuntary memory even with high cue-overlap, that is, reduced degree of priming.

### Method

#### Participants

Forty-six participants (28 females, mean age = 27.64, *SD* = 6.95, range = 19 to 49, 23 per group) were recruited from the Medical Research Council Cognition and Brain Sciences Unit Volunteers Panel (see online supplemental materials). Eligibility criteria were (a) aged 18 to 65, (b) reported no history of mental health, neurological or psychiatric illness, (c) had not participated in related studies, (d) able to attend two laboratory sessions one week apart, and (e) willing to complete a pen-and-paper diary. Participants provided their written and informed consent prior to the study, after being informed of the potentially distressing nature of the film. They were also reminded that they could withdraw from the study at any point. Approval for all experiments was obtained from the University of Cambridge Psychology Research Ethics Committee (2014/3214). Based on an effect size of *d* = .91 from Holmes et al. (2009), 23 participants per group allowed for more than 80% probability of detecting a significant group difference on diary intrusions (α = .05, two-tailed).

#### Materials

##### Trauma film

This was a 12-min film using multiple (rather than single) clips. It comprised 11 different discrete scenes depicting injuries, violence, and death, and each with unique topic content (same as that used in Holmes et al., 2009; James et al., 2015). The scene clips were from sources such as government road traffic safety adverts, documentary footage, and news footage. The content included, for example, scenes of an elephant on a rampage, a man injuring himself by cutting his throat, and an eye operation. These clips have been used previously in both behavioral (Deeprose et al., 2012; Holmes et al., 2009; James et al., 2015) and neuroimaging studies (Bourne, Mackay, & Holmes, 2013; Clark, Holmes, Woolrich, & Mackay, 2016; Reiser et al., 2014) to successfully generate intrusions (see online supplemental materials). The film was played via E-Prime Version 2.0 (Schneider, Eschman, & Zuccolotto, 2002) and viewed on a desktop screen (size: 32 cm × 40 cm; resolution:1280 × 1024 pixels; distance: 100 cm approximately from the screen). Audio was played from headphones.

##### Cognitive interference task: Film reminder cues plus Tetris

*Film reminder cues.* These comprised 11 stills—one from each of the discrete scenes from the film—presented one at a time against a black background for 3 sec using E-Prime Version 2.0 (Schneider et al., 2002). These stills typically depicted the instance before the worst moments, which have been clinically associated with intrusive memories (Ehlers, Hackmann, & Michael, 2004). These included, for example, a picture of a circus (before the elephant escapes and goes on a rampage) and a smiling teenager (just before he was hit by a van while being distracted by texting). Participants were instructed to “sit still and pay close attention to the pictures.” The stills were presented in the same fixed order as the corresponding scenes within the film.

*Tetris.* A desktop-based version of Tetris (Blue Planet Software, 2007) was used. This computer game used seven 2D geometric blocks of different shape and color, which fall from the top of the screen, one at a time. Each block can be rotated 90 degrees at a time using the arrow keys on the computer keyboard. The game’s objective is to form full horizontal lines using the blocks without leaving any gaps; points are awarded each time a full line is completed. To encourage the use of mental rotation (Iyadurai, Blackwell, et al., 2018; James et al., 2015; Lau-Zhu et al., 2017), participants were instructed to pay attention to the three blocks appearing in the preview at the top right of the screen, which were due to fall after the one being played. They were told to use their mind’s eye to work out the best way to manipulate and place the blocks to achieve a line. The game was adaptive with individual’s performance (i.e., becoming more difficult as participants’ scores increased). Tetris was played in marathon mode (with 15 levels) and with the sound off. We did not collect data on performance—ways to measure performance are limited in the scoring constraints of this commercial game (e.g., scoring is not linear and there are scoring rules, such as for certain pieces, which are hard to interpret). However, note that higher Tetris scores in this game have been associated with fewer intrusions (James et al., 2015) and higher visuospatial WM capacity (Lau-Zhu et al., 2017).

##### Filler tasks

This 30-min structured break consisted of performing a knowledge search task twice, separated by a music filler task (as used in Deeprose et al., 2012; Holmes et al., 2009; Holmes, James, et al., 2010). See the online supplemental materials for further details.

##### Self-report measures

Baseline measures assessed for depressive symptoms (Beck, Steer, & Brown, 1996), trait anxiety (Spiel-berger, Gorsuch, Lushene, Vagg, & Jacobs, 1983), prior trauma history (Foa, Ehlers, Clark, Tolin, & Orsillo, 1999), and general use of mental imagery (Nelis, Holmes, Griffith, & Raes, 2014). Additional manipulation checks with self-reported ratings were performed in line with our previous work (e.g., James et al., 2015; James, Lau-Zhu, Tickle, et al., 2016), to assess negative mood before and after watching the film, the amount of attention paid to the film and personal reference of the film, compliance with completing the diary, and expectation on task manipulation. See the online supplemental materials for further details on these measures.

#### Measures of memory of the trauma film

These varied in retrieval intention (involuntary vs. voluntary retrieval) and degree of cue overlap (high vs. low). All (i.e., except the diary) were presented using MATLAB R2009a (The MathWorks Inc., 2009) and Psychtoolbox (Brainard, 1997).

##### Intrusion diary

In a pen-and-paper tabular diary (Deeprose et al., 2012; Holmes et al., 2009; Holmes, James, et al., 2010; James et al., 2015), participants were asked to note down their intrusions over a 1-week period after film viewing. Both verbal and written instructions were given on how to complete the diary. An intrusive memory was defined as “visual images, sounds and bodily sensations related to the film” and that “pop into mind without one expecting it”; such images could range from “fuzzy and fragmented to vivid and as clear as normal vision”. They were told not to include memories of the film that were retrieved deliberately. The diary was split by days, and within each day into three time periods (morning, afternoon and evening). Participants were instructed to keep the diary with them, and note down the intrusion (in a tick box) as soon as it occurred within the corresponding period, and also any associated trigger cues they could have identified. For each intrusion, they wrote down a brief description (e.g., an image of the eye operation) that was later used to verify whether the intrusion was indeed from the film or not. Participants were also asked to set aside regular times for each period to review the diary and encouraged to note down ‘0’ if no intrusions occurred in that period. The main outcome was the total count of intrusive image-based memories. Because intrusion rates on individual days are typically low, our main outcome was the total number of intrusive image-based memories summed across a 1-week period (Deeprose et al., 2012; Holmes et al., 2004, 2009; James, Lau-Zhu, Clark, et al., 2016). This measure was deemed to index involuntary retrieval with low cue-overlap (i.e., relative to recognition and priming tasks).

##### Free-recall task

The instructions and the scoring system from the Autobiographical Interview (AI; Levine, Svoboda, Hay, Winocur, & Moscovitch, 2002) were adapted to free recall of the trauma film (see the online supplemental materials for further details). The AI has been shown to have high interrater reliability (0.88 to 0.96) for scoring autobiographical memories, real-life traumatic memories in PTSD (McKinnon et al., 2015), and memories of nontrauma film footage (St-Laurent, Moscovitch, Jadd, & McAndrews, 2014). Detailed written instructions were presented on the screen to guide recall and participants were instructed to verbalize their responses using tape recorder. There were two recall phases. First (free recall), participants were instructed to retrieve as many details as possible from the film; they were told to recall the clips in any order and were allowed a 10-min period. No additional retrieval cues were given at this stage. Afterward (specific probing), participants were given cue phrases for each of the 11 scenes in a randomized fixed order, and were allowed a 2-min period for each scene to retrieve additional details.

Verbalizations were subsequently transcribed and followed a process of text segmentation into details—meaningful units of information (Levine et al., 2002). Nonepisodic content was not counted, such as general opinions and comments in relation to other events (e.g., “these things shouldn’t happen to people”). Accurate episodic details were identified, meaning details that pertained directly to what actually took place in the film (St-Laurent et al., 2014), and further categorized into either *event* or *perceptual* details. The main outcome was the total number of episodic details. This task was deemed to index voluntary retrieval with low cue-overlap (relative to recognition and priming).

##### Priming task

**Figure 3:**
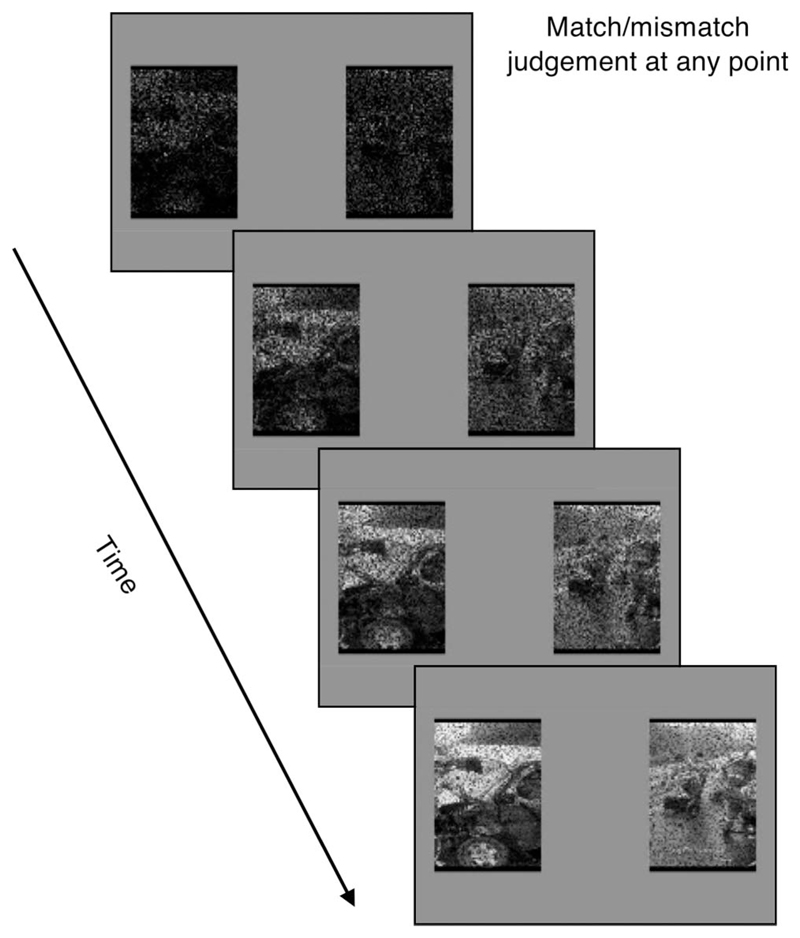
Schematic of a trial in the priming task in Experiment 1. Participants were presented with still-halves and were asked to judge whether or not both halves matched—that is, whether both halves belonged to the same original still. The still-halves were initially covered by salt-and-pepper noise (black and white pixels superimposed on the still), and became progressively clearer over 6 sec, as 20% of the noise pixels were removed every 1250 msec. The fully revealed still-halves remained on screen for up to 2 sec further. Participants could make a response at any point in these 8 sec (either with some noise or fully clear), with the trial terminating upon a response. This figure is for illustration and thus not to scale. Stimuli in the actual experiment were in color (not black-and-white).

The stimuli consisted of two sets of 90 stills (different to the stills used as film reminder cues). One set contained stills drawn from the trauma film; another set contained foil stills selected based on similarity to the film stills in content and themes (i.e., death and injury). Each still was split along the midline, producing two still-halves (for a schematic see Figure 3; see the online supplemental materials for further details). In a given trial, two still-halves were presented simultaneously. These still-halves, when put together, could either recompose into the same original still (75% of trials—match response), or be from completely unrelated stills (25% of trials—mismatch response). Participants were asked to judge whether the two still-halves were a match or a mismatch. There were a total of 144 trials. Pairings of still-halves for each trial were fixed, and the order of the trials was randomized.

Each trial started with a fixation cross in the middle of a gray screen for 2 sec, followed by the still-halves. A continuous identification paradigm was applied (Berry, Shanks, & Henson, 2008): the still-halves were initially covered by salt-and-pepper noise (black and white pixels superimposed on the still), and then became progressively clearer over 6 sec, as 20% of the noise pixels were removed every 1250 msec. The fully revealed still-halves remained on screen for up to 2 sec further. Participants could make a response at any point in these 8 sec (either with some noise or fully clear), with the trial terminating upon a response.

We reasoned that reaction time (RT) to still-halves would be faster (i.e., decisions made at higher levels of noise) for trials with stills of the trauma film than trials with foil stills. This would occur even though no reference was made to prior exposure to films (i.e., participants would show perceptual priming), rendering this task an indirect measure that is unlikely to involve voluntary retrieval (Richardson-Klavehn & Bjork, 1988). The main outcome was RT for accurate trials. This task was deemed to index involuntary retrieval with high cue-overlap (akin to recognition).

##### Recognition task

This task used the same two still sets as in the priming task. There were 180 trials. In each, a still was presented for up to 5 sec and participants were asked to judge whether or not (yes/no response) each still belonged to the trauma film, as fast and as accurately as possible. After each still, participants were also asked to provide a confidence rating for each response made using a scale from 1 (*pure guess*) to 4 (*extremely confident*) within 5 sec, with the trial ending upon a response. Trial order was randomized across participants. This measure was deemed to index voluntary retrieval with high cue-overlap.

#### Procedure

##### Session 1

See Figure 2 for schematic overview. On Day 1, after providing written and informed consent, participants completed baseline self-report measures and practiced playing Tetris for 3 min. Afterward, they completed mood ratings prior to watching the film. They then watched the film alone; they were asked to imagine they were bystanders witnessing the scenes. Following film viewing, they completed mood ratings again, and additional ratings on attention to film and personal relevance of the film. All participants then had a 30-min break completing filler tasks.

After the break, participants were randomly allocated to one of two groups. Participants in the reminder-plus-Tetris group performed the interference task with both components: they were shown the film reminder cues, and then played Tetris for 10 min. Participants in the reminder-only group were given the film reminder cues and then asked to sit quietly for 10 min. Trauma film, film reminder cues and Tetris were all presented on the same desktop screen. At the end of the session, participants were given detailed verbal and written instructions on completing the diary.

##### Session 2

At the follow-up session a week later (Day 8), participants returned the diaries and then completed computer-based memory tasks (on same screen as in Session 1) in the following fixed order: recall (free recall and specific probing), priming, and recognition. They then completed ratings on demand and diary compliance. Finally, they were debriefed and reimbursed for their participation.

#### Statistical analyses

Data were examined for potential univariate outliers within each condition (>3 *SD* from the mean; Tabachnick & Fidell, 1996) following previous studies using similar paradigms (e.g., Deeprose et al., 2012; Holmes et al., 2004), but none were found. For the relevant memory tasks, performance above chance was assessed using one-sample *t* tests. Between-groups comparisons were conducted using independent sample *t* tests, with homogeneity of variance assessed using Levene’s statistic. Analyses of variance (ANOVA) with repeated measures were used when both within-group and between-groups variables were included (i.e., for group comparisons between memory tasks/time points), with sphericity assumptions assessed using the Mauchly’s test statistic. If assumptions of parametric tests were violated, corresponding nonparametric tests were applied. When patterns of results converged across tests, only results from the parametric tests were reported. A two-tailed alpha level of .05 was used unless stated otherwise. When indicated, we also used a Bayesian approach to check whether there was sufficient evidence to support the null—the absence of group differences (see the online supplemental materials). Analyses were performed using SPSS Version 25.0 (IBM Corp., 2013).

### Results

Groups also did not significantly differ in any baseline measures, mood ratings or task manipulation checks (see the online supplemental materials). Below we first present group effects within each memory task and then across tasks.

#### Effects of the cognitive interference task on each memory task

##### Intrusion diary (Days 1 to 7)

**Figure 4:**
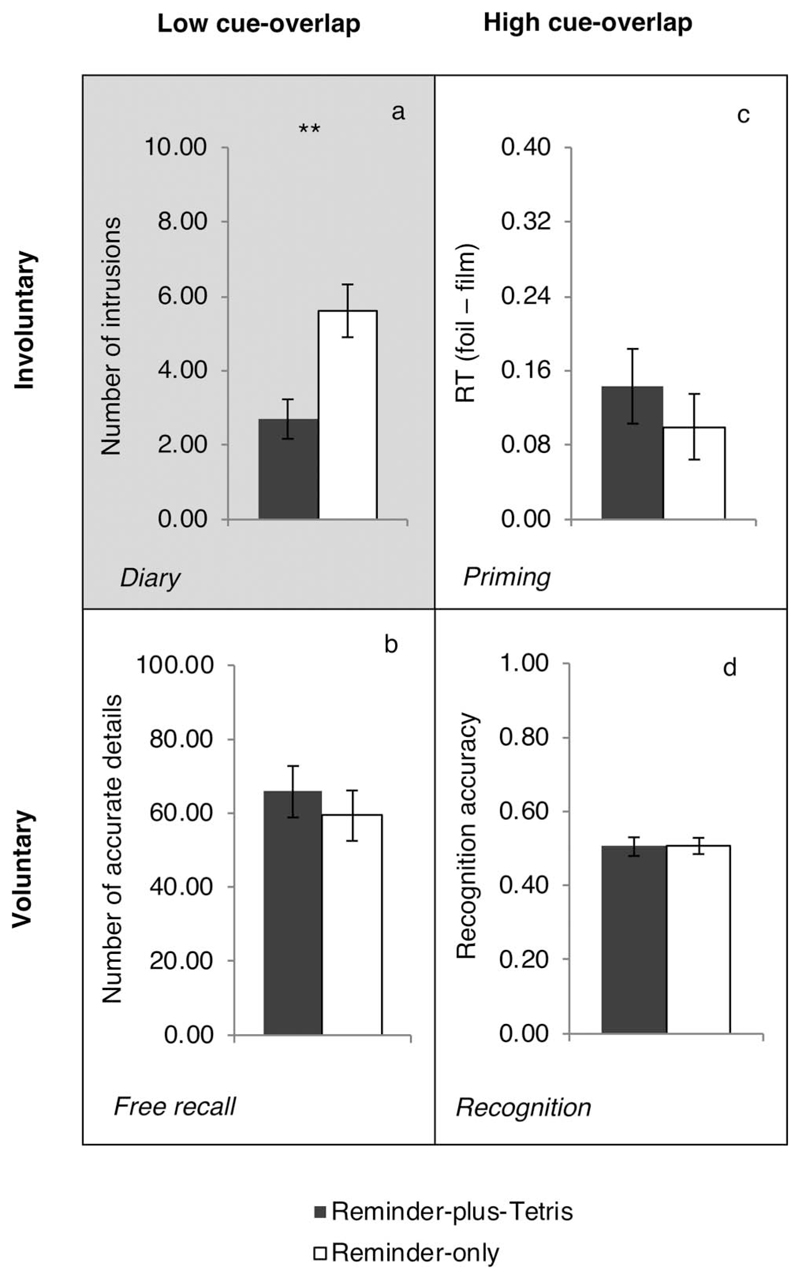
Main results from Experiment 1 by group for each memory task: (a) intrusion diary (involuntary with low cue-overlap), (b) free recall (voluntary with low cue-overlap), (c) priming (involuntary with high cue-overlap), and (d) recognition (voluntary with high cue-overlap). Error bars represent ±1 *SEM*. **Significant two-tailed group comparisons within each task (*p* < .01)—only for intrusion diary (cell highlighted with gray background for emphasis).

All diaries were checked and rated for the numbers of intrusive memories by two researchers independently. Interclass correlation (two-way mixed effects model, consistency, single measure; McGraw & Wong, 1996) was 1.00, suggesting full agreement. Eighty-seven percent of reported intrusive memories were matched to scenes of the film, suggesting the majority were of the experimental trauma (others were excluded from further analysis). Overall, the mean number of intrusions was 4.15 (*SD* = 3.31; range = 0–14), similar to previous studies (Deeprose et al., 2012; Holmes et al., 2009; James, Lau-Zhu, Tickle, et al., 2016). Further, the majority of intrusions (80.1%) were reported to be associated with cues in everyday life (see the online supplemental materials). Critically and as predicted, the reminder-plus-Tetris group reported significantly fewer intrusive memories over the week compared with the reminder-only group, *t*(44) = 3.29, *p* = .002, *d* = .97, 95% CI of *d* [0.34, 1.56] (Table 1 and Figure 4).

##### Recognition task (Day 8)

Each trial was classified as a hit (correct identification of film still), miss (incorrect identified of film still), false alarm (FA; incorrect identification of foil still), or correct rejection (CR; correct identification of foil still; Table 1). Recognition accuracy score for each participant was calculated by subtracting the FA rate (FA/[FA + CR]) from the hit rate (hit/[hit + miss]). Positive accuracy scores indicated that memory performance was above chance, which was the case for both groups, *t*s(22) > 20.03, *p*s < .001, *d*s > 4.17 (see Figure 4). However, there was no significant group difference in recognition accuracy, *t*(44) = 0.05, *p* =.959, *d* < .01, 95% CI of *d* [蜢0.58, 0.58]. Also see the online supplemental materials.

##### Priming task (Day 8)

A priming index was calculated for each participant by subtracting the mean RT for film trials from the mean RT for foil trials across match and mismatch trials (see Table 1). Positive priming scores would indicate that film stills were more quickly and correctly identified than foil stills, which was the case in both groups, *t*s(22) > 2.83, *p*s < .05, *d*s > .59, suggesting that perceptual priming occurred (see Figure 4). Critically, there was no significant group difference in the degree of priming, *t*(44) =0.81, *p* =.420, *d* =.22, 95% CI of *d* [蜢0.80, 0.36].

##### Free-recall task (Day 8)

All individual scripts were scored based on the procedure adapted from the original AI (Levine et al., 2002). A subsample of 22% of these scripts (10 of 46) was selected at random and rescored by another researcher. Interclass correlations (two-way mixed effects model, consistency, single measures; McGraw & Wong, 1996) for the free recall stage were 0.96 for event details, 0.69 for perceptual details, and 0.97 for both combined, and for the specific probing stage were 0.90 for event details, 0.90 for perceptual details, and 0.88 for both combined. Therefore, almost all coding showed excellent agreement, whereas coding for perceptual details during free recall showed good agreement (Cicchetti, 1994). There was no significant group difference in the total number of episodic details (event and perceptual) during *free* recall, *t*(44) =0.67, *p* =.510, *d* =.20, 95% CI of *d* [ 0.77, 0.39] (see Figure 4). There were also no significant group differences if the analyses were conducted separately on event and perceptual details, *t*s < 1, or by including additional details prompted by specific probing, *t*s < 1 (see Table 1).

#### Comparing retrieval intention and cue overlap

The lack of significant effects on the three memory tasks (apart from the diary) could simply be type II errors. To explicitly test whether there were significant effects of the retrieval intention and/or of cue overlap on the degree of interference, we combined all four tasks into a single ANOVA. To enable comparison across tasks, we standardized the main outcome from each memory task (*z* scored across all participants, i.e., in both groups). These four outcomes were: number of diary intrusions, number of accurate details at free recall, priming RT index and recognition accuracy. A 2 (between-groups: reminder-plus-Tetris and reminder-only group) ȕ 2 (within-group: involuntary and voluntary) ȕ 2 (within-group: high and low cue-overlap) mixed model ANOVA on these *z*-scores revealed that none of the main effects, *F*s < 1, nor the two-way interactions were significant: group =intention, *F*(1, 44) = 2.17, *p* =.148, group ȕ cue-overlap, *F*(1, 44) = 3.15, *p* = .083, and intention ȕ cue-overlap, *F* < 1. Critically, the three-way interaction between group ȕ intention ȕ cue-overlap was significant, *F*(1, 44) = 6.89, *p* = .012, $\eta_{p}^{2}=.135.$

The above three-way interaction was decomposed into subsequent 2 ȕ 2 ANOVAs on each level of the third variable. The analysis using 2 (groups) ȕ 2 (cue overlap) ANOVA showed that the group ȕ cue-overlap interaction was significant for tasks of involuntary memory (diary vs. priming), *F*(1, 44) = 7.60, *p* =.008, $\eta_{p}^{2}\text{ = .147}$, but not for tasks of voluntary memory (recall vs. recognition), *F* < 1. Further, the analysis using 2 (groups) ȕ 2 (intention) ANOVA showed that the group ȕ intention interaction was significant for tasks with low cue-overlap (diary vs. recall), *F*(1, 44) = 9.78, *p* =.003, $\eta_{p}^{2}\text{ = .182}$, but not for tasks with high cue-overlap (priming vs. recognition), *F* < 1. Taken together, these analyses confirmed that the interference effect on intrusions was significantly larger than on free recall and priming. These results converge to suggest that interference was selective to diary intrusions (see Figure 4).

### Discussion

Experiment 1 investigated, for participants who viewed a trauma film, the effect of performing an interference task (following a film reminder cue) 30 min after the film on the subsequent memory of that film. Memory was assessed by a battery of measures that differed in retrieval intention (involuntary vs. voluntary) and cue overlap (low vs. high). Confirming our first prediction, and replicating previous studies (Holmes et al., 2009; Holmes, James, et al., 2010), the reminder-plus-Tetris group reported fewer intrusive memories in the diary (involuntary memory with low cue-overlap) than the reminder-only group, whereas no significant group differences were found in accuracy on a recognition task (voluntary memory with high cue-overlap).

Regarding the novel hypothesis about the role of cue overlap, there were no significant differences between the reminder-plus-Tetris group and reminder-only group for the new memory tasks, namely, free recall (voluntary with low cue-overlap) and priming (involuntary with high cue-overlap). Indeed, a significant three-way interaction supported the inference that there was interference only on the number of intrusions (in line also with analyses using a Bayesian approach; see the online supplemental materials). These findings suggest that cue overlap (at least as operationalized in this experiment) cannot explain the interference effect. Nor can involuntary retrieval alone, as interference was not observed on all involuntary measures. Thus, a combination of involuntary retrieval and low cue-overlap appears necessary to explain the interference effect, and/or the intrusion diary differs from the other three memory tasks along some other dimension (as explored in Experiments 2 and 3 later).

There were no interference effects on free recall, even though (as with the intrusion diary) it lacked copy cues from the trauma film (like those provided for the recognition task). As noted in the Introduction, this is not to deny that some types of cue were present to trigger the diary intrusions outside the laboratory. Indeed, participants reported that diary intrusions were triggered by everyday (external/environmental) cues (see online supplemental materials), consistent with the broad literature on involuntary autobiographical memories (Berntsen, 1996, 1998, 2009, 2010; Berntsen, Staugaard, & Sørensen, 2013; Conway, 2001; Staugaard & Berntsen, 2014) and clinical research on intrusive memories (Ehlers & Clark, 2000; Michael et al., 2005). It is also possible that the potential for cue-memory overlap is broad (Vannucci et al., 2015), so that everyday cues triggering diary intrusions do not necessarily have lower cue-overlap. Nonetheless, if the key to an interference effect were only the combination of some type of retrieval cue (whether copy or not, which is present even for diary intrusions) and involuntary recall, then we should have observed an interference effect in priming, which we did not. Thus, we reasoned that another dimension in relation to cue processing (beyond cue overlap) ought to be considered, which can better account the selective interference. We addressed one possibility in Experiment 2, where we directly assessed the degree of attentional capture by retrieval cues (as well as providing those cues in a better-controlled laboratory assessment of intrusions, in the form of a novel vigilance-intrusion task).

Although the use of different memory tasks in the current experiment was mainly to manipulate cue overlap/retrieval intention, these tasks also provide additional theoretical information. Free recall, for example, provided some further methodological advantages in relation to recognition tasks. Recognition memory is thought to involve both *recollection* of episodic information and a nonepisodic feeling of *familiarity* (Yonelinas, 2002), where the latter might arise from recent activation of parts of semantic memory. One could argue that the interference task disrupts recollection (episodic details) but not familiarity, such that recognition performance in the reminder-plus-Tetris group was preserved because of an intact familiarity process. The lack of interference on our free-recall task rules out this possibility. We isolated episodic (event and perceptual) content in the freely recalled transcripts by adapting a standardized method (Levine et al., 2002; McKinnon et al., 2015; St-Laurent et al., 2014), and were still unable to find an interference effect. The lack of interference on recollection processes is further supported by the absence of group differences in additional exploratory analyses on recognition performance, either by confidence ratings in Experiment 1, or also by remember and know judgments in Experiment 2 (see online supplemental materials).

Our lack of interference effect on priming may be at odds with some clinical accounts. Enhanced perceptual priming of trauma stimuli has been theorized to underline later intrusion development (Ehlers & Clark, 2000; Holz, Lass-Hennemann, Streb, Pfaltz, & Michael, 2014; Sündermann, Hauschildt, & Ehlers, 2013), and also affect the long-term perceptual memory system governing intrusive symptoms according to the dual representation theory (Brewin, 2014). Instead, we found a reduction in intrusion rates despite an apparent lack of interference effects on priming. We return to such broader theoretical implications in the General Discussion.

An unaddressed confound is the different in delay interval between film watching and completing the different memory tasks. The diary score was summed over Days 1 to 7 after the film (to obtain enough intrusions for statistical analyses), whereas the scores on the other three measures were all acquired on Day 8. It is possible that the interference effect is short-lived, affecting retrieval early on (e.g., for a few days after encoding) but not later (e.g., a week after encoding), which would produce the current pattern of results. When we attempted to match the delay across all memory measures in a post hoc analysis—by restricting the diary data to just Day 7 (see online supplemental materials)—the critical three-way interaction (i.e., bigger interference effects on diary intrusions than on other measures) was no longer significant. However, we think this is likely to reflect unreliable estimates of intrusion rates, given the low number of intrusions on a single (final) day in the diary (for which the average number of intrusions in the reminder-only group was less than one; see the online supplemental materials). Further, the selective interference effect has already been demonstrated even when both assessments of recognition and intrusions were matched on delay (i.e., both assessed on Day 8 in the laboratory; and using an intrusion provocation task), albeit when a postencoding interference was 24 h after the trauma film (James et al., 2015). Nevertheless, we also attempted to assess intrusion and voluntary memory with better-matched delays in Experiment 2.

Finally, in a fixed-order design as ours, it is possible that delivery of one memory measure may have contaminated later ones. For example, a group difference in an earlier memory measure might spill over to cause an artifactual group difference in subsequent measures. This was not the case in our experiment, because the intrusion diary (the first measure administered) showed a group difference, but the subsequent measures did not. It is also possible that the reverse contamination happens, such that a group difference in one measure (e.g., intrusion diary) masks a real group difference in subsequent measures, for example, by promoting rehearsal (Ball, 2007; Mace, 2014). To help address this possibility of order effects, we included measures of intrusions both *before* and *after* other memory measures in the next experiment.

## Experiment 2: Attentional Capture

Selective interference on diary intrusions in Experiment 1—but not on any of the other measures of memory—suggests that neither the diary’s involuntary aspect, nor its low cue-overlap (at least in terms of lacking copy cues relative to the recognition task using film stills), can fully account for the interference effect. The main aim of Experiment 2 was to investigate an alternative possibility, namely that interference disrupts the ability of external cues to capture attention, thereby reducing access to the memory (see Figure 1). To take an example from an intrusion diary: having a red vehicle pass by—that is similar in some respects to what was seen in the trauma film—may attract the person’s attention and trigger an intrusion, even though that vehicle was not originally the focus of attention (e.g., because that person was working at a cafe). When those cues are already the center of attention (as in the recognition or priming task in Experiment 1), there may not be scope for an interference effect to be revealed. Our consideration of attentional capture also chimes with the wider literature linking preferential processing of trauma/threat-related cues with the development of stress-related psychopathologies (Mathews & MacLeod, 2005; Öhman, Flykt, & Esteves, 2001), including intrusive symptoms (Ehlers & Clark, 2000; Michael & Ehlers, 2007; Sündermann et al., 2013; Verwoerd, Wessel, de Jong, & Nieuwenhuis, 2009). Attentional capture is typically thought as automatic (involuntary) and nonconscious, so one may not always be aware of potential cues (Ehlers & Clark, 2000). To investigate the role of attentional capture in explaining the interference effects, we directly measured the degree of attentional capture using a novel adaptation of the dot-probe task (MacLeod, Mathews, & Tata, 1986; see Methods for further details).

The second aim of Experiment 2 was to address the potential confounds of both retrieval delay and order of the measures, which may have affected the results of Experiment 1. To enable this, we assessed intrusions within the laboratory (Lau-Zhu, Holmes, & Porcheret, 2018; Takarangi, Strange, & Lindsay, 2014), devising a method we call the *vigilance-intrusion* task, based on a go/no-go paradigm (see the Method section for further details). Intrusions here occur in the context of a task (albeit low-demanding)—rather than during rest (as in James et al., 2015)—so opportunities for contamination from voluntary retrieval might be reduced (Lau-Zhu et al., 2018). Because this task furnished a sufficient number of intrusions in a short timeframe (10 min), we were able to administer it twice: on Day 1, immediately before the attentional-capture task, and on Day 8, immediately before the recognition task (see Figure 2). This design helped improve match in delay (both intrusion and recognition assessed on Day 8) and account for order effects (intrusions assessed before *and* after attentional capture). It also allowed us to explore whether interference on intrusions varies depending on delays (e.g., immediately vs. a week later).

### Hypotheses

Replicating Experiment 1, we predicted that the reminder-plus-Tetris group would have fewer diary intrusions (Days 1–7) than the reminder-only group, but show comparable performance on recognition (Day 8; i.e., the selective interference effect). We also predicted fewer intrusions in the reminder-plus-Tetris group for the new vigilance-intrusion task, at least on Day 8, which would replicate that pattern of intrusion/recognition dissociation on Day 8 found by James et al. (2015). Novel to this experiment, we predicted that, if the interference task affects the ability of cues to attract attention, then the reminder-plus-Tetris group would show reduced attentional capture to trauma-film cues (see the Method section), in parallel to reduced intrusion rates. The importance of this retrieval factor in explaining access to the memory trace would be more consistent with single-trace accounts, without the need to invoke separate-trace accounts (see Figure 1).

### Method

#### Participants

Thirty-six participants took part in the experiment (19 females, mean age = 25.67, *SD* = 7.06, age range = 19 to 49, 18 per group). The same recruitment strategy as in Experiment 1 was used (see the online supplemental materials). This sample size gave 81% power to detect the interference effect of *d* = .97 on the number of diary intrusions in Experiment 1 (α = .05; two-tailed).

#### Materials

All materials and stimuli were identical to Experiment 1, with the exception of the following measures of memory. See the online supplemental materials for further details.

#### Measures of memory of the trauma film

The intrusion diary was identical to Experiment 1. So was the recognition task, except that participants provided remember/know judgments instead of confidence ratings (see the online supplemental materials). All memory tasks (except the intrusion diary) were presented using MATLAB R2009a (The MathWorks Inc., 2009) and Psychtoolbox (Brainard, 1997).

##### Vigilance-intrusion task

This was adapted from the Sustained Attention to Response Task (SART; Murphy, Macpherson, Jeyabalasingham, Manly, & Dunn, 2013; Robertson, Manly, Andrade, Baddeley, & Yiend, 1997). It comprised 11 film stills and 68 foil stills: film stills were drawn from the trauma film and were similar in content to the film reminder cues; foil stills depicted a variety of colored indoor/outdoor scenes. All stills were altered using Gaussian Blur 2.0 (thus were not exact replicas of the film). This blurring procedure was intended to emulate cues glimpsed in daily life when they are outside of one’s focus of attention (Berntsen, 2009), and was used previously in another laboratory-based intrusion paradigm (James et al., 2015; James, Lau-Zhu, Tickle, et al., 2016; Lang et al., 2009).

Participants were asked to perform a vigilance task with 270 trials. Each trial started with a centrally presented digit (1 to 9) on a black background screen for 250 msec (see the online supplemental materials). The digit then disappeared, and the black screen remained for a further 1500 msec. Participants were instructed to press the ‘Go’ key using the desktop keyboard for digits between ‘1’ to ‘9,’ but withhold their response for ‘3’ (occurring 11% of the time). Every three trials starting from the first, a foil still appeared behind the digit (instead of a black background). Participants were told that, in addition to the digits, they may also encounter background scenes, but no responses to the scenes were required. Both digits and scene stills were presented in a fixed randomized order.

**Figure 5:**
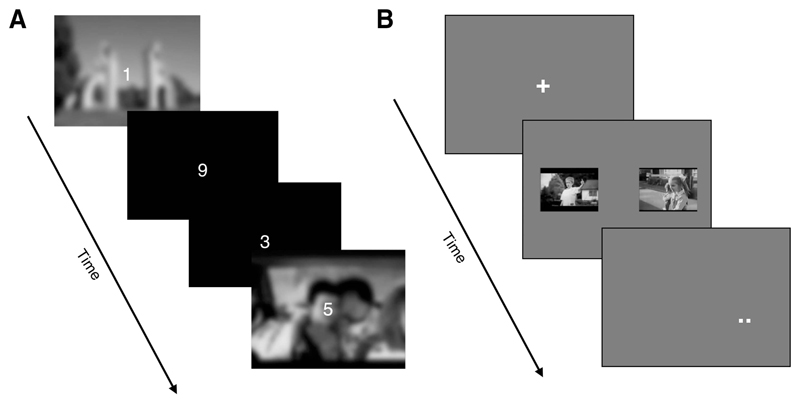
Schematic of memory tasks in Experiment 2. Sample trials of the vigilance-intrusion task are presented in panel A. In each trial, a digit was centrally presented. Participants were instructed to press the GO key every time they saw a digit that was not ‘3,’ and to press the Intrusion key whenever they experienced an intrusive memory of the film. This task is also used in Experiment 3 albeit with slight modifications. A sample trial of the attentional-capture task is presented in panel B. Participants were presented with a film-foil still pair, which quickly disappeared and was followed by a dot probe behind the original location of either still. Participants were instructed to judge the identity of the dot probe (i.e., one or two dots) as accurately and as quickly as possible. Pictures are for illustration only and thus not to scale. Stimuli in the experiment were in color (not black-and-white).

Participants were told that intrusive memories from the film (using the same definition of intrusions as used with the diary) might pop up spontaneously at any time during the vigilance task. In that case, they were instructed to press the Intrusion key using the keyboard to pause the vigilance task and note down a brief description of the intrusion’s content (so it could be later verified as with the diary). They then resumed the vigilance task by pressing a button on the keyboard to complete any remaining trials. Task duration was around 9 min (but time was added when participant paused to record an intrusion). Viewing distance was 60 cm approximately from the screen. The main outcome was the total number of intrusive memories throughout the vigilance task. See Figure 5a for an illustration of the task.

Attentional-capture task: This was adapted from the dot-probe task by MacLeod et al. (1986). The stimuli consisted of two sets of 96 stills, one set for the trauma films and the other for foils (as described for the priming task in Experiment 1). For each set, half of the stills were categorized as emotional stills and half as neutral stills (based on a negative emotionality index obtained from independent norming on participants who had not seen the trauma film). The task had four runs with 96 trials using the entire stimulus set per run. A trial consisted of a pairing between a film and foil still matched on emotionality ratings.

Each trial began with a central fixation cross for 1000 msec followed by the still pair for either 500 msec or 1000 msec. Each still appeared to the left and right of the cross, respectively. The still pair then disappeared, and a small visual target (a dot probe) was presented in the location where one of the stills was shown. Participants were asked to judge as quickly and as accurately as possible whether the target had one or two small dots. Each dot subtended at a visual angle of 0.10 ȕ 0.10 degrees approximately (see the online supplemental materials). The trial terminated upon response. An error-triggered delay message appeared for every mistake (for 5 sec) before participants moved on to the next trial. The location of each still type was randomized across trials. Specific pairings between stills were randomized across participants. The background color remained dark gray throughout the task. Viewing distance was approximately 60 cm from the screen. The main outcome was *attentional bias* toward film stills over foil stills, as expressed by the degree to which the speed of correct target discrimination was quicker when the target was presented in the location shared with the film still rather than with the foil still. See Figure 5b for an illustration of the task.

##### Procedure

###### Session 1

See Figure 2 for a schematic overview. On Day 1, all procedures remained identical to Experiment 1 up to random allocation to either the reminder-plus-Tetris group or the reminder-only group. Then, after a short practice (see the online supplemental materials), participants completed the vigilance-intrusion task. Afterward, they performed the attentional-capture task. Finally, instructions on completing the intrusion diary were given.

###### Session 2

At the follow-up session a week later (Day 8), participants gave back their diaries. They then completed the vigilance-intrusion task (same as in Session 1), followed by the recognition task. Finally, they were debriefed and reimbursed for their participation.

##### Statistical analyses

Data were examined for potential univariate outliers as in Experiment 1. Three outliers were identified (one for the reminder-plus-Tetris group on intrusion frequency in the vigilance-intrusion task on Day 1, one for the reminder-plus-Tetris group on intrusion frequency in the diary, and one for the reminder-only group on recognition accuracy), and these were changed to one unit larger (if the score was below the mean) or smaller (if the score was above the mean) than the next most extreme score in the distribution (Tabachnick & Fidell, 1996). Pearson product–moment correlation was used to assess the linear relationship between two variables. Otherwise, the statistical methods were identical to those in Experiment 1.

#### Results

Groups also did not significantly differ in any baseline measures, mood ratings or task manipulation checks, except with diary compliance (see online supplemental materials). Adding diary compliance as a covariate into the relevant analyses did not change the pattern of results. Below we first present group effects within each task/time point and then across tasks/time points.

##### Effects of the cognitive interference task on each memory task

###### Intrusion diary (Days 1 to 7)

The total number of intrusive memories in all diaries were checked and counted by two researchers independently. Interclass correlation (two-way mixed effects model, consistency, single measure; McGraw & Wong, 1996) was 0.98, suggesting near perfect agreement. Ninety-eight percent of all intrusions were matched to scenes of the film, suggesting that the majority were of the laboratory experience (others were excluded from further analysis). Overall, the mean number of intrusion was 5.61 (*SD* = 1.29; range = 0–24), also similar to previous studies (Deeprose et al., 2012; Holmes et al., 2009; James et al., 2015). Similar to Experiment 1, the majority of intrusions (70.3%) were reported to be associated with a cue in everyday life (see online supplemental materials). As predicted, the reminder-plus-Tetris group reported significantly fewer diary intrusions compared with the reminder-only group, *t*(34) = 3.69, *p* = .001, *d* = 1.23, 95% CI of *d* [0.49, 1.91] (see Table 2), in line with Experiment 1.

###### Memory tasks on Day 8: Intrusions and recognition

*Recognition task (Day 8).* Recognition accuracy was scored using the same procedure as in Experiment 1 (see Table 2). Recognition accuracy was above chance in both groups, *t*s(17) > 13.51, *p*s < .001, *d*s > 3.18. There was no significant group difference in recognition accuracy between the reminder-plus-Tetris group (*M* = 0.46, *SD* = 0.10) and the reminder-only group (*M* = 0.42, *SD* = 0.13), *t*(34) = 1.07, *p* = .292, *d* = .34, 95% CI of *d* [蜢1.00, 0.32] (also see the online supplemental materials).

*Vigilance-intrusion task (Day 8).* The majority of laboratory intrusions (98%) were matched to the trauma film (others were excluded from further analysis). Overall, the mean number of intrusion was 7.14 (*SD* = 5.65; range = 0–24), which was higher than in James et al. (2015; mean of 3–4 intrusions), where a different/shorter (2-min) laboratory assessment was used (also see the online supplemental materials). Critically and as predicted, the reminder-plus-Tetris group reported significantly fewer laboratory intrusions than the reminder-only group on Day 8, *t*(34) = 2.42, *p* = .021, *d* = .81, 95% CI of *d* [0.11, 1.47] (see Table 2).

*Comparing intrusions and recognition on Day 8.* We ran a 2 (between-groups: reminder-plus-Tetris and reminder-only) ȕ 2 (within-group: intrusion and recognition) mixed model ANOVA on standardized scores (*z* scores) to equate the vigilance-intrusion task and the recognition task (both on Day 8) on the same metric. The critical group ȕ intention interaction was significant, *F*(1, 34) = 7.06, *p* = .012, $\eta_{p}^{2}\text{ = .172}$, confirming that there were significant group differences in intrusions but not recognition, even when both measures were better matched on delay (i.e., one week after the trauma film).

###### Memory measures on Day 1: Intrusions and attentional bias

**Figure 6:**
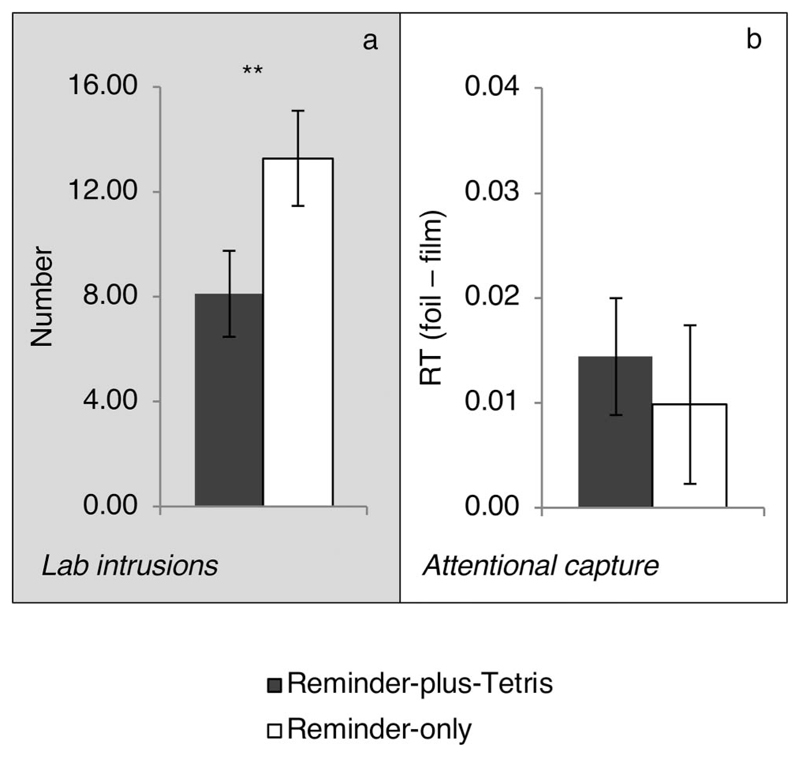
Experiment 2: Tasks assessing memory of the trauma film by group on Day 1: (a) vigilance-intrusion task and (b) attentional-capture task (results restricted to bias for ‘emotional’ film stills). Error bars represent ±1 *SEM*. **Significant two-tailed group comparisons within each task (*p* < .01)–only for a: vigilance-intrusion task (cell highlighted with gray background for emphasis).

*Vigilance-intrusion task (Day 1).* The majority of all laboratory intrusions (99%) were matched to scenes of the film, in line with the same task on Day 8 (others were excluded from further analysis). Overall, the mean number of intrusion was 10.25 (*SD* = 6.95) and the range was 0 to 28. The numbers of these early intrusions were predictive of diary intrusions and of laboratory-intrusions on Day 8 (see online supplemental materials). Critically, the reminder-plus-Tetris group reported significantly fewer intrusions than the reminder-only group on the vigilance-intrusion task also on Day 1, *t*(34) = 2.87, *p* = .007, *d* = 0.96, 95% CI of *d* [0.25, 1.62] (Table 2 and Figure 6), replicating the pattern on Day 8.

*Attentional-capture task (Day 1).* The proportion of correct trials was equivalent between groups, *t* < 1 (see Table 2). RTs were obtained from all correct trials with RT <2000 msec (Hoppitt et al., 2014; See, MacLeod, & Bridle, 2009). Attentional-bias scores were calculated for each participant according to still emotionality type, by obtaining the RT difference for responding to targets sharing location with foil stills versus targets sharing location with trauma film stills. Positive scores indicated a faster response—thus a bias—for trauma film stills. Such a trauma-film bias was significant within each group (one-tailed) for emotional still-pairs only, *t*s(17) > 1.80, *p*s < .090, *d*s > .44, but not neutral still-pairs, *t*s(17) < 0.39, *p*s > .701 (see Table 2), suggesting that attentional capture was pronounced for film cues depicting emotional content. Nevertheless, there was no significant group differences in attentional bias to trauma-film cues (of emotional scenes), *t*(34) = 0.61, *p* = .545, *d* = .16, 95% CI of *d* [蜢0.85, 0.46] (see Figure 6). Also see the online supplemental materials.

*Comparing intrusions and attentional capture on Day 1.* The lack of a group difference on attentional biases was unexpected, given that we found a group difference on intrusions assessed during a similar time period (i.e., soon after interference on Day 1). Therefore, we directly compared the interference effect on intrusions versus attentional bias. As with Experiment 1, a single outcome was selected from each memory task and compared using standardized *z* scores in the same analysis (*z* scored across all participants, i.e., in both groups). We selected the number of early laboratory-intrusions on the vigilance-intrusion task, and the attentional-bias score to trauma film stills (across both emotional and neutral still pairs). A 2 (between-groups: reminder-plus-Tetris and reminder-only) ȕ 2 (within-group: early intrusions and attentional capture) mixed model ANOVA revealed that there were no main effects of group, *F*(1, 34) = 3.45, *p* = .072, or of memory task, *F* = 1. The group = memory measure interaction also failed to reach significance, *F*(1, 34) = 3.93, *p* = .055. When we repeated this analysis by considering attentional-bias score to emotional trauma-film scenes only (as the bias was mainly evident for trials with emotional still-pairs), the main effects of group, *F*(1, 34) = 1.95, *p* = .172, and of memory task, *F* < 1, continued to be nonsignificant, but now the group = memory measure interaction was significant, *F*(1, 34) = 6.34, *p* = .017, $\eta_{p}^{2}\text{ = .157}$. Figure 6 shows that group differences were more pronounced for laboratory intrusions than for attentional capture (to emotional trauma film scenes).

##### Discussion

We tested whether the interference task reduces intrusive memories via a reduction in attention capture—the ability of film-related cues in the environment to capture attention. If so, then we expected that, alongside an interference effect on intrusions, an interference effect would also be revealed on the degree of attentional capture to trauma-film cues (measured by RTs in the adapted dot-probe task). This new task was sensitive enough to detect an attentional bias to trauma-film cues relative to matched foil stills that had not been seen before (provided those stills depicted emotional scenes of the trauma film). However, there were no significant group differences in the size of this attentional capture, despite a significant group difference in the number of laboratory intrusions assessed within the same period (Day 1). Indeed, a combined (*z* scored) analysis showed a significant interaction in the direction of a greater interference effect on intrusions relative to the degree of attentional capture (also see the online supplemental materials for analyses using a Bayesian approach). Importantly, the interference effect on intrusions remained even though intrusions were assessed before (in the vigilance-intrusion task on Day 1) *and* after (in the diary and the vigilance-intrusion task on Day 8) the attentional-capture task within our overall procedure (see Figure 2), addressing the potential task-order confound of Experiment 1 where intrusions were assessed only first. Hence, these findings suggest that the degree of attentional capture by potential retrieval cues is unlikely to explain the discrepancy between intrusions and other memory measures in neither Experiment 2 (recognition) nor Experiment 1 (recall, recognition and priming), despite potential attentional differences between measures.

The lack of association between intrusions and attentional capture may be at odds with research linking attentional biases and stress-related psychopathology (Ehlers & Clark, 2000; Mathews & MacLeod, 2005; Michael & Ehlers, 2007; Öhman et al., 2001; Sündermann et al., 2013; Verwoerd et al., 2009). Note, however, that our attention-capture task used copy cues of the event, unlike other types of cues in past studies (e.g., words or noncopy pictures). Thus, intrusions and attentional bias may still be related through other measures/domains, and other manipulations may be able to reduce intrusion rates via changes in attentional capture (Verwoerd, Wessel, & de Jong, 2012; Verwoerd et al., 2009), but these do not seem to apply to the current selective interference effect.

Experiment 2 provided further confirmation of the selective interference on intrusions while sparing voluntary memory. We found that the reminder-plus-Tetris group reported fewer intrusions than the reminder-only group according to (a) a 1-week diary, replicating Experiment 1 as well as previous studies (e.g., Holmes et al., 2009, 2010; James et al., 2015), (b) a vigilance-intrusion task performed on Day 8 (replicating James et al., 2015), and (c) a vigilance-intrusion task on Day 1 (novel to this experiment). Yet the groups showed equivalent recognition performance. The greater number of intrusions provided by the vigilance-intrusion task (relative to diary) also meant that we could directly compare measures within similar period (Day 8)—as in James et al. (2015)—addressing the potential confound in Experiment 1 where intrusions and recognition were assessed at different delays after the trauma film. Moreover, both measures were further matched by both being assessed within the laboratory context, whereas in most studies to date they have been assessed in different contexts (i.e., the diary being conducted in daily life; Lau-Zhu et al., 2018). A combined analysis on Day 8 also showed a significantly greater interference effect on laboratory intrusions than recognition performance. Together, this pattern of findings reinforces the claim that the intrusion/recognition dissociation is indeed genuine, despite not being predicted by single-trace memory theories. Therefore, what remains critical—besides continuing to demonstrate this involuntary/voluntary dissociation—is to identify what retrieval factors modulate the size of the interference effect on intrusions per se (as we attempt in Experiment 3).

An intriguing finding—established for the first time here—is that the impact of the interference task on intrusions could be observed early on, just *minutes* after the intervention was carried out (within the same laboratory session as film viewing and interference). These findings suggest that the interference effect is both immediate and long-term, despite alternative claims that emotional memory effects only emerge at longer delay intervals, for example, after consolidation has taken place (e.g., Dudai, 2004; McGaugh, 2004; Nader et al., 2000). We return to this issue in the General Discussion. Furthermore, variations in early intrusions also predicted the number of intrusions in the subsequent week-long diary across groups (see the online supplemental materials). Hence for now we have established that the vigilance-intrusion task administered within the first laboratory session can serve as an analogue for a subsequent 1-week diary. This allows for single-session experiments without the need for participants to return at a later date (Lau-Zhu et al., 2018; Takarangi et al., 2014), and obviates the potential burden of keeping a 1-week diary. We therefore exploited and extended the vigilance-intrusion task in Experiment 3.

A potential concern is that participants who experienced more intrusions (i.e., the reminder-only group) necessarily paused the vigilance-intrusion task more often to provide intrusions’ descriptions. One might wonder whether more pausing also allowed more time to be spent on, for example, ruminating about the films, which in turn could have inflated the intrusion rates in the reminder-only group. We addressed this concern in Experiment 3 by removing the need to verbally describe intrusions, given that we already confirmed here that participants can indeed correctly identify intrusive memories of the film.

One may also wonder why attentional capture was not assessed within the vigilance-intrusion task, and/or why intrusions were not assessed within the attentional capture (dot-probe) task, to maximize comparability. The vigilance-intrusion task involved a low-demand task which results in performance levels close to ceiling, presumably providing little room to simultaneously measure any attentional capture (since its purpose was to occupy participants just enough to minimize opportunities for voluntary retrieval). The dot-probe task, by contrast, needed to be sufficiently challenging to measure attentional capture, which might be compromised if participants were additionally required to report intrusions concurrently. Nevertheless, future experimental adaptations may enable simultaneous measurement of intrusions and other forms of attentional capture (e.g., Barzykowski & Niedźwieńska, 2018; Vannucci, Batool, Pelagatti, & Mazzoni, 2014). Instead, however, we tested the remaining retrieval factor identified in the General Introduction (see Figure 1) in the next experiment, namely whether the level of retrieval load modulated the interference effect.

### Experiment 3: Retrieval Load

Given that Experiments 1 and 2 suggest that neither cue overlap nor attentional capture are able to explain the interference effect on intrusions, the main aim of Experiment 3 was to investigate the role of retrieval load (see Figure 1)—specifically the possibility that the interference effect is unique to retrieval contexts with low cognitive-demands (henceforth low *retrieval-load*) and absent (or smaller) in contexts with higher cognitive-demands. Note that load here refers to load during *retrieval* (i.e., while memory is being assessed) and not at other time points (e.g., the load imposed by Tetris game-play to presumably disrupt consolidation). As alluded to before, the main difference between the vigilance-intrusion task and the attentional-capture task was that the first involved a monotonous task (i.e., low retrieval-load), whereas the second emphasized speed and accuracy with performance feedback (i.e., high retrieval-load), which may have left fewer resources for a memory trace to be activated (e.g., for intrusions to emerge). This chimes with evidence that involuntary autobiographical memories are more likely to be elicited during low-demanding tasks inducing a diffused state of attention (Berntsen, 2009; Schlagman & Kvavilashvili, 2008) than during high-demanding tasks (Ball, 2007; Barzykowski & Niedźwieńska, 2018; Vannucci et al., 2015). One could also argue that the priming task in Experiment 1 and the voluntary-memory tasks in Experiments 1 and 2 entailed higher retrieval-load than the everyday tasks during which intrusions occurred according to the diary (see Figure 1).

To test the retrieval load hypothesis in Experiment 3, we manipulated load levels during the vigilance-intrusion task (that was validated in Experiment 2) by using concurrent WM tasks. Participants performed three times a novel version of the vigilance-intrusion task, each time with a different (within-group) load condition: no load, visuospatial load (additional visuospatial WM task), and verbal load (additional verbal WM task). The contrast between verbal and visuospatial WM tasks allowed us to test whether a potential lack of (or smaller) interference effect in retrieval conditions with high load depends on the load’s modality. We expected that an additional visuospatial WM load would leave less room for intrusive memories, given claims that visuospatial WM shares modality-specific resources (Andrade, Kavanagh, & Baddeley, 1997; Baddeley & Andrade, 2000) and neurocircuitry (Albers, Kok, Toni, Dijkerman, & de Lange, 2013; Pearson, Naselaris, Holmes, & Kosslyn, 2015) with visual imagery, which appears to underlie many intrusive memories in clinical populations (Ehlers et al., 2004; Grey & Holmes, 2008; Hackmann, Ehlers, Speckens, & Clark, 2004; Holmes, Grey, & Young, 2005). However, it is also possible that any (even verbal) WM load (e.g., by taxing general-domain central executive functions) reduces the opportunity for intrusions (Engelhard et al., 2010; Gunter & Bodner, 2008; van den Hout & Engelhard, 2012), thereby reducing the sensitivity to an interference effect.

Note that unlike in Experiments 1 and 2 where the nature of intrusive memories was inferred indirectly (i.e., by comparing intrusion tasks with other memory tasks that did not involve intrusion monitoring), here we tested the effect of concurrent load levels (and their interaction with the interference effect) *directly* on intrusions rates.

In addition to addressing potential contributions of retrieval factors to the selectivity of the interference effect, it is also important to establish which aspects of the interference procedure are required to produce the interference effect itself. This is an important methodological issue for future research wishing to investigate/replicate this selective interference effect, and for translational applications (e.g., whether it is necessary to remind a victim of their recent trauma before intervening with an interference task). Thus, we also sought to establish whether *both* components of our interference procedure (film reminder cues and Tetris game-play) are needed to produce the interference effect. As already alluded in the General Introduction, our previous studies (including current Experiments 1 and 2) have all used reminder cues when an interference task was performed 30 min after the film (Deeprose et al., 2012; Holmes et al., 2009; Holmes, James, et al., 2010)—with the rationale that the cues help orient attention to the target event (Visser et al., 2018)—but the necessity of such reminder cues in this timeframe remain untested (unlike evidence that such cues are indeed needed 24 h after the film; Experiment 2 in James et al., 2015). We tested the requirement for a reminder cue by adding a third group of participants who played Tetris without such cue (*Tetris-only* group).

#### Hypotheses

First, we predicted a replication of the key finding from Experiment 2 showing that the reminder-plus-Tetris group experience fewer laboratory intrusions relative to the reminder-only group, using the same vigilance-intrusion task with *key presses*. A novel hypothesis concerned the effects of retrieval load on intrusions in the vigilance-intrusion task, using a modified version where participants retrospectively reported the number of intrusions they experienced—henceforth the vigilance-intrusion task with *estimates* (see Methods for rationale). We hypothesized that the interference effect would be modulated by (interact with) retrieval load, such that the reminder-plus-Tetris group would have fewer intrusive memories than the reminder-only group when there is low retrieval-load during intrusion retrieval, but this interference would be absent (or at least smaller) when there is high retrieval-load instead (especially if that load involves visuospatial WM). Finally, if the interference effect on intrusions is conditional upon a reminder cue prior to the interference task, then the reminder-plus-Tetris group would show fewer intrusions memories than both the reminder-only group and the new Tetris-only group.

#### Method

##### Participants

Fifty-seven participants took part in this experiment (34 females, mean age = 26.88, *SD* = 6.75, age range = 18 to 45, 19 per each of the three group; see the online supplemental materials). The same recruitment strategy was used as in Experiments 1 and 2. This sample size provided a power of 82% to replicate an interference effect of *d* = 0.96 on the number of laboratory intrusion on the vigilance-intrusion task on Day 1 in Experiment 2 (α = .05; two-tailed).

##### Materials

All materials were identical to Experiment 1 and 2, except for the additional modifications to the vigilance-intrusion tasks.

##### Vigilance-intrusion tasks

There were two versions (with either key presses or estimates), both presented using MATLAB R2009a (The MathWorks Inc., 2009) and Psychtoolbox (Brainard, 1997); see Figure 5.

###### Vigilance-intrusion with key presses

This version was identical to the one in Experiment 2, except that there was no longer the requirement to pause the task to provide a written description for each intrusion. Pressing the Intrusion key did not pause the vigilance task, thus the duration of this task was the same for all participants (i.e., 9 min). This version with online reporting was included to maximize our ability to replicate the interference effect on early intrusions in Experiment 2 (Stage I; see the Procedure section), in case such an effect was moderated by reporting method (e.g., because of possible underestimation of intrusion rates using retrospective recall, as below).

###### Vigilance-intrusion with estimates

Additional vigilance-intrusion tasks were administered with further modifications to test the retrieval load hypothesis (Stage II; see the Procedure section). Critically, there was no longer the need to press the Intrusion key when participants experienced an intrusion. Instead, intrusions were assessed using retrospective estimates (Schaich, Watkins, & Ehring, 2013; Zetsche, Ehring, & Ehlers, 2009). The original design (270 trials) was divided into three consecutive runs (three 3-min runs with 90 trials each). As background scenes, each run presented each of the 11 film stills once, alongside 19 foil stills (different from those presented in the vigilance-intrusion task with key presses). After each run, the task paused so that participants could estimate how many intrusions they had for that run (*how often did memories of the event in the form of mental images pop into your mind in the last three minutes?*) by typing in the corresponding count using the number keypad on the keyboard (see the online supplemental materials for further details). We reasoned that retrospective recall bias would be minimized compared with giving a single rating for a full 9-min period. The total number of intrusions per condition was summed across the three consecutive 3-min runs.

The use of estimates after 3-min runs, and removing the need for key presses to report intrusions on the fly, meant that participants could more readily perform the vigilance-intrusion task and a WM task simultaneously, allowing for our intended manipulation of retrieval load. Otherwise, they would have had to perform three tasks simultaneously (vigilance, WM task and intrusion reporting with key presses). Importantly, participants performed the digit-vigilance task using their nondominant hand (and the Mouse rather than the keyboard), freeing up their dominant hands required for one of the WM tasks described below.

###### WM tasks

These tasks served as additional (within-group) loads to the latter version of the vigilance-intrusion task. A finger-tapping task was used as the additional visuospatial WM load (Baddeley, 2003; Baddeley & Andrade, 2000). This involved tapping a pattern using a square box with a 5 ȕ 5 array of buttons (Bourne et al., 2010; Deeprose et al., 2012; Holmes et al., 2004). Each button was labeled with an individual letter from A to Y, running from left to right. Participants had to tap an irregular pattern of five keys (*JYPVA*). They were encouraged to visualize the pattern in their mind’s eye while tapping steadily. A counting-backward task was used as the additional verbal WM load (Baddeley, 2003; Baddeley & Andrade, 2000). This involved counting backward aloud in 1s, beginning from a number seed (e.g., starting from 969 and continuing to 968, 967, etc.). Participants were encouraged to count steadily. The no load condition involved neither of these tasks.

##### Procedure

See Figure 2 for a schematic overview. There was a single laboratory session. All procedures remained identical to Experiments 1 and 2 up to random allocation to one of the three groups: reminder-plus-Tetris, reminder-only, or Tetris-only. Participants in the latter group played Tetris for 10 min without prior exposure to film reminder cues.

All participants performed all vigilance-intrusions tasks. In Stage I, the vigilance-intrusion task (with *key presses*) was completed to replicate key findings on Experiment 2 on early laboratory-intrusions using online reporting.

In Stage II, additional vigilance-intrusion tasks were completed to test the retrieval load hypothesis. This stage was further divided into two phases (training and experimental). In the training phase, participants were familiarized with the modified version of the vigilance-intrusion task to use retrospective to estimate intrusion rates, and also practiced the WM memory tasks. For finger tapping, participants overpracticed this task by tapping the sequence for 5 min without interruption, with the tapping box out of sight and without visual feedback (similar to Holmes et al., 2004). For counting, participants were asked to count backward for 5 min without feedback.

In the experimental phase, participants completed the vigilance-intrusion task (with *estimates*) under all three conditions of WM loads in an counterbalanced order (controlling for both effects of load order and time). For each load condition, three consecutive 3-min runs were completed. For no load, the vigilance-intrusion task was performed as it is. For visuospatial load, participants began each run of the vigilance-intrusion task with a reminder to tap the visuospatial pattern, and were asked to stop tapping at the end of a run. Tapping responses were recorded by the computer program. For the verbal load, participants began each run of the vigilance-intrusion task with predesignated number seeds (958, 845, and 969, respectively, as in Deeprose et al., 2012) alongside a reminder to start counting out loud, and were asked to stop counting at the end of a run. Their verbal responses were tape-recorded. Finally, participants were debriefed and reimbursed.

##### Statistical analyses

Data were examined for potential univariate outliers as in Experiments 1 and 2. One outlier (for the reminder-plus-Tetris group on intrusion frequency in the vigilance-intrusion task with estimates, no load condition) was identified and changed to one unit smaller than the next most extreme score in the distribution (Tabachnick & Fidell, 1996). Otherwise, the statistical methods were identical to those in Experiments 1 and 2. For comparability with Experiments 1 and 2, below we present results in a similar fashion: (a) group effects within each vigilance-intrusion task followed by group effects across task versions; (b) all analyses were restricted to the two main groups (reminder-plus-Tetris and reminder-only) unless otherwise indicated; analyses with all three groups (i.e., including the additional group Tetris-only) did not change the pattern of results.

#### Results

Groups also did not significantly differ in any baseline measures, mood ratings or task manipulation checks (see the online supplemental materials).

##### Effects of the cognitive interference task on laboratory intrusions

###### Vigilance-intrusion with key presses

This initial version of the task provided a direct replication of the key findings from Experiment 2 (except that participants did not pause the task to describe intrusions). Overall, the mean number of intrusion was 15.54 (*SD* = 11.56; range = 0–56), which was higher than in Experiment 2. Replicating the pattern from Experiment 2, the reminder-plus-Tetris group (*M* = 9.37, *SD* = 8.48) reported significantly fewer early laboratory-intrusions, as indicated simply by intrusion key-presses, compared with the reminder-only group (*M* = 21.11, *SD* = 10.98), *t*(36) = 3.69, *p* = .001, *d* = 1.20, 95% CI of *d* [0.48, 1.86].

###### Vigilance-intrusion with estimates

All groups showed equivalent performance for the finger-tapping task and the counting-backward task (see the online supplemental materials). In the *no-load* condition, the mean number of intrusion was 12.40 (*SD* = 9.92; range = 0蜢50), slightly lower than the task version using key presses. Below we first present group effects per retrieval-load condition and then across conditions.

**Figure 7:**
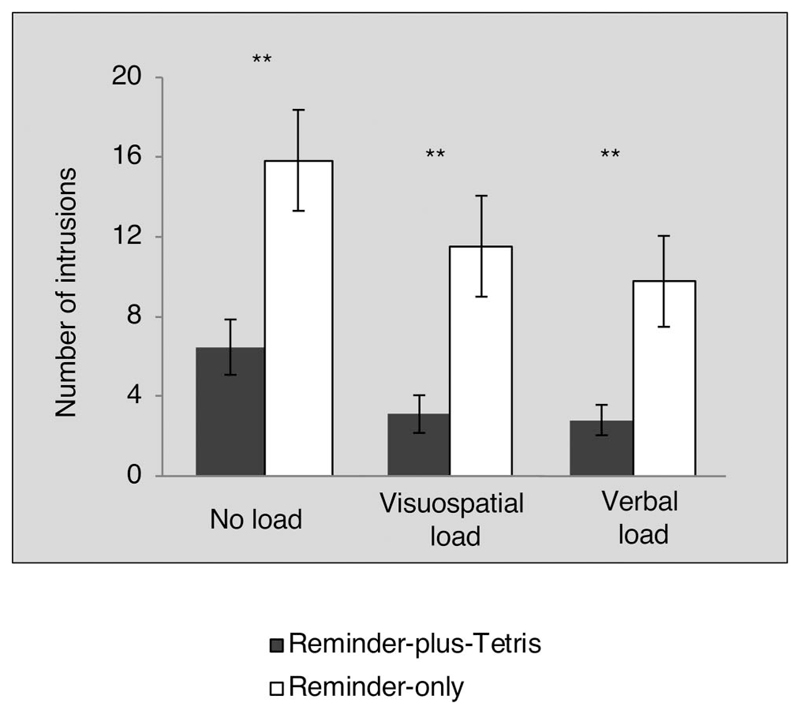
Experiment 3: Number of laboratory intrusions by group and type of retrieval load within the vigilance-intrusion task with estimates. Error bars represent ±1 *SEM*. The Tetris-only group was not included for comparability with Experiments 1 and 2. **Significant two-tailed pairwise group comparisons within each retrieval load (** *p* < = .01)–all retrieval-load conditions (cells were all highlighted with gray background for emphasis, for comparability with previous figures on selective interference on intrusions).

The reminder-plus-Tetris group reported significantly fewer intrusions compared with the reminder-only group, in the *no-load* condition, *t*(36) = 3.24, *p* = .003, *d* = 0.77, 95% CI of *d* [0.35, 1.71], in the *visuospatial-load* condition, *t*(36) = 2.66, *p* = .014, *d* = 0.86, 95% CI of *d* [0.17, 1.50], as well as in the *verbal-load* condition, *t*(36) = 2.89, *p* = .008, *d* = 0.84, 95% CI of *d* [0.25, 1.59] (see Figure 7).

To directly compare the *sizes* of the interference effect in the three load conditions, we ran a 2 (between-groups: reminder-plus-Tetris and reminder-only) ȕ 3 (within-group: no, visuospatial and verbal retrieval load) mixed model ANOVA. As expected, this analysis yielded a main effect group, *F*(1, 36) = 12.46, *p* = .001, $\eta_{p}^{2}\text{ = .257}$, confirming that the reminder-plus-Tetris group (*M* – 4.25, *SE* = 1.60) estimated significantly fewer intrusions overall relative to the reminder-only group (*M* = 12.32, *SE* = 1.60, *p* = .001) across all conditions. There was also a significant main effect of retrieval load, *F*(2, 72) = 7.22, *p* = .001, $\eta_{p}^{2}\text{ = .167}$. To unpack this load effect, post hoc comparisons showed that relative to no load (*M* = 11.16, *SE* = 1.44), there were significantly fewer intrusions during visuospatial (*M* = 7.45, *SE* = 1.50; *p* < .006) and verbal retrieval-load (*M* = 6.24, *SE* = 1.19; *p* < .002), but no significant differences between the latter two (*p* = .358). The critical group ȕ retrieval-load interaction, however, was not significant, *F* < 1. This suggests that, contrary to expectations, the interference effect on intrusions did not vary according to the level of retrieval load during the vigilance-intrusion task, nor according to the modality of retrieval load (visuospatial or verbal; see Figure 7).

###### Necessity of reminder cues prior to interference task

Our final aim was to investigate whether the reminder cue is needed prior to Tetris game-play to interfere with intrusions. These analyses included all three groups and sought convergence across two forms of intrusion reporting. We ran a 3 (between-groups: reminder-plus-Tetris, reminder-only and Tetris-only) ȕ 2 (within-group: key presses or estimates during the no load condition) mixed ANOVA on the number of intrusions. This revealed a significant main effect group, *F*(2, 54) = 7.29, *p* = .002, $\eta_{p}^{2}\text{ = .212}$, for which post hoc tests indicated (a) the expected finding that the reminder-plus-Tetris group (*M* = 7.92, *SE* = 2.02) reported significantly fewer intrusions than the reminder-only (*M* = 18.47, *SE* = 2.02, *p* = .001), (b) critically that the reminder-plus-Tetris group *also* reported fewer intrusions than the Tetris-only group (*M* = 15.53, *SE* = 2.02, *p* = .010), and (c) there were no significant group differences between the reminder-only and Tetris-only (*p* = .306). The pattern of findings remained even after applying Bonferroni corrections (α = .017). Overall, it appears that only the combination of reminder cues and Tetris leads to reduction in intrusions.

There was also a significant main effect of intrusion reporting-method, *F*(1, 54) = 6.56, *p* = .013, $\eta_{p}^{2}\text{ = .108}$, suggesting that key presses (*M* = 15.54, *SE* = 1.42) were associated with more intrusions than retrospective estimation (*M* = 12.40, *SE* = 1.21), but the group ȕ reporting method was not significant, *F* < 1. Thus, retrospection may underestimate intrusion rates but still be sensitive enough to reveal the interference effect (as in the analyses above).

##### Discussion

Experiment 3 again replicated the interference effect on intrusions in a vigilance-intrusion task (with key presses), even when intrusions were reported at fixed task-duration (a previous confound in Experiment 2). Critically, the degree of interference did not vary significantly according to whether participants were engaged in a concurrent verbal or visuospatial WM load during a new version of the vigilance-intrusion task (with estimates). These results therefore fail to support the hypothesis that interference on intrusions is absent (or smaller) when participants are taxed by high retrieval-load. We hypothesized that (visuospatial/verbal) retrieval load during the vigilance-intrusion task would compete with the resources needed for intrusions to occur, leaving less room for an interference effect. Although manipulations of both visuospatial and verbal load (compared with no load) at intrusion retrieval did reduce intrusion rates overall, neither of these retrieval load effects interacted with group, and interference was detected in all three load-conditions. In other words, retrieval load appears detrimental to intrusions, consistent with research on in voluntary memories (Ball, 2007; Barzykowski & Niedźwieńska, 2018; Berntsen, 2009; Schlagman & Kvavilashvili, 2008; Vannucci et al., 2015), but such effects appear to be additional and independent from the effects exerted at the time of intervention by the interference task (Tetris after reminder cues). This finding that yet another obvious retrieval factor—here retrieval load—does not appear to explain the interference effects on (intrusive) memory is difficult to reconcile with single-trace accounts (see Figure 1). We return to the broader theoretical implications in the General Discussion.

The equivalent reduction in intrusive memories by a concurrent visuospatial versus verbal load is consistent with a general-load effect (Engelhard et al., 2010; Gunter & Bodner, 2008; van den Hout & Engelhard, 2012) rather than modality-specific effects (Andrade et al., 1997; Baddeley & Andrade, 2000; Bourne et al., 2010; Brewin, 2014; Holmes et al., 2004; Holmes, James, et al., 2010; Lau-Zhu et al., 2017). However, the load effects in Experiment 3 concern (intrusive) memory as experienced *during* a WM-load manipulation (Engelhard et al., 2010; Leer et al., 2017; van den Hout, Eidhof, Verboom, Littel, & Engelhard, 2014), whereas previous research supporting a modality-specific account mostly concern (intrusive) memory as experienced *after* a WM-load manipulations (for a review, see James, Lau-Zhu, Clark, et al., 2016). Future research could systematically manipulate modality and load levels, while also assessing intrusions both during and following WM loads, to delineate the impact and time course of modality-specific versus general-load effects (also see the online supplemental materials).

Intrusion rates were reduced only when Tetris was preceded by a reminder cue (i.e., not by Tetris alone), here 30 min after the film. As we have reasoned previously, many other types of information can enter WM during a 30-min period after an experience; an orientation cue might be important in allowing the target memory to be brought into attention sufficiently for interference to be exerted (Visser et al., 2018). For this reason, we have also used a cue before gameplay in the first hours after real trauma while patients are waiting in hospital in a different context to the one in which the trauma occurred, namely accidents on the road (Iyadurai, Blackwell, et al., 2018). Hence, the reminder cue appears to be a procedural requirement to bring about the selective interference effect in future studies.

Critically, the third group in Experiment 3 provided additional theoretical leverage. One could have argued that reminder cues in the initial control group (reminder-only group in Experiments 1–3) led to retrieval practice during the 10-min silence period, which then *increased* intrusions above the reminder-plus-Tetris group, rather than the latter group showing *reduced* intrusions per se. The inclusion of the Tetris-only group here served as an additional active control-group, ruling out a potential reminder-boosting effect. Specifically, the Tetris-only group showed number of intrusions comparable with the reminder-only group, suggesting that the reminder cues in themselves in the reminder-only group were unlikely to have increased intrusion. Hence, the additional Tetris-only group is not only relevant for replications/translations, but also strengths our interpretation from Experiments 1 and 2 that the interference task *reduces* intrusive memories.

Experiment 3 did not directly compare intrusive versus voluntary memory. The finding that load during memory assessments fail to moderate the interference effect suggests that retrieval load is unlikely to have been a critical confound in previous demonstrations of the intrusive/voluntary memory dissociation (including those in Experiments 1 and 2). However, high load in recognition tasks is only assumed. Future replications could compare both intrusive and voluntary memories while directly manipulating (and measuring) retrieval load within both memory conditions.

#### General Discussion

Three experiments assessed the impact of an interference task (film reminder cues followed by Tetris game-play)—delivered after encoding of a film with traumatic content—on intrusive (involuntary) versus voluntary memory of the film. Although trauma film research over the last two decades has revealed that interference tasks can affect intrusive but not voluntary memory, this has never been shown while systematically controlling for key methodological differences between the two types of memory retrieval, as we did here using a battery of novel memory measures (see Figure 2). We first summarize our key findings, and then discuss their theoretical implications for the controversial debate concerning the relationship between involuntary (intrusive) and voluntary memory (also see the Introduction). We argue that our findings challenge single-trace memory theories, and further constrain separate-trace memory theories (see Figure 1). We conclude with general methodological limitations and possible future directions.

##### Summary of Findings

Key findings are presented in Figures 4, 6, and 7. The interference task reduced the number of intrusive memories in a 1-week diary (Experiments 1 and 2; Figure 1), but did not impact performance on well-matched measures of voluntary retrieval, namely free recall (Experiment 1; Figure 4) and recognition (Experiments 1 and 2; Figure 4) at one week. Moreover, neither did the interference task impact other measures of involuntary retrieval, namely perceptual priming by film cues (Experiment 1; Figure 4), nor attentional capture by film cues (Experiment 2; Figure 6).

However, we were able to extend the interference effect on intrusions recorded in a diary to intrusions reported in a laboratory assessment (the vigilance-intrusion task). Different intrusion assessments furnished different rates of intrusions. From highest to lowest intrusion rates, intrusions were assessed by vigilance-task on Day 1 using key presses (Experiment 3); with retrospective estimations (Experiment 3); additional validating reports (Experiment 2); vigilance-task on Day 8 (Experiment 2); and finally diary intrusions (Experiments 1 and 2). Vigilance-intrusions tasks not only produced higher intrusion rates, but also within a shorter timeframe and within the same laboratory context and time point as the other measures of memory, providing further match to those measures. Yet, all intrusion reporting-methods were sufficiently sensitive to reveal interference. Interference effects on laboratory intrusions were observed on Day 8 (Experiment 2), soon after interference on Day 1 (Experiments 2 and 3; Figure 6), and irrespective of the degree and type of WM load at retrieval (Experiment 3; Figure 7).

We can also more confidently interpret our overall findings as the interference task (reminder-plus-Tetris) *reducing* intrusions, as opposed to the reminder cues in the control group (reminder-only) *increasing* intrusions; otherwise, the latter would have boosted intrusions against an additional active control group without such cues (Tetris-only), but that was not the case (Experiment 3).

Taken together, our new battery of memory measures suggests that the apparent dissociation between intrusive and voluntary memory is not accounted for by the most obvious retrieval factors, as informed by foundational textbook theories of memory (Baddeley et al., 2009) and key accounts of involuntary memory (Berntsen, 2009), namely cue overlap (Experiment 1; Figure 4), attentional capture (Experiment 2; Figure 6), and retrieval load (Experiment 3; Figure 7). Importantly, neither were our findings explained by group differences in baseline measures, measures for film viewing, task compliance nor expectations (see the online supplemental materials). This would seem difficult to reconcile with single-trace theories, and more compatible with separate-trace theories in which intrusions arise from a memory system separate to that underlying (voluntary) episodic memory (see Figure 1). Our data therefore extend a considerable number of previous claims that interference tasks impact intrusions while sparing voluntary expressions of the memory (Bourne et al., 2010; Brewin, 2014; Brewin & Saunders, 2001; Deeprose et al., 2012; Holmes et al., 2004, 2009; Holmes, James, et al., 2010; James et al., 2015; Krans et al., 2010).

##### Theoretical Implications

Single-trace theories broadly propose a single system underlying episodic memory (Squire & Zola-Morgan, 1991; Tulving, 1972, 2002) and autobiographical memory (Berntsen, 2009; Conway, 2001; Conway & Pleydell-Pearce, 2000; Rubin et al., 2008). These theories generally assume that the same memory trace is accessed for involuntary and voluntary conscious retrieval of episodes. Therefore, any differential effects of the interference task on intrusions versus voluntary memory are likely to arise at the time of retrieval—because of methodological differences between the various memory tasks—rather than genuine differences in the underlying memory trace. If so, by matching or controlling for such retrieval factors, we should cease to observe the selective interference effect, that is, no longer see a differential impact on involuntary versus voluntary retrieval (Experiments 1 and 2), or at least be able to modulate the size of the interference effect on intrusions (Experiment 3). However, when we manipulated the three obvious retrieval factors (see Figure 1), as informed by core textbook memory principles (Baddeley et al., 2009; Berntsen, 2009), we found that interference remained selective to intrusive memories, and regardless of retrieval context. It is possible that there is yet another retrieval factor that is critical and that we did not explore, but until then, the present data seem difficult to reconcile with single-trace accounts in which interference disrupts the same trace involved in intrusions and voluntary retrieval.

Our data are more consistent with separate-trace accounts of memory that permit distinct traces for intrusive and voluntary memory (see Figure 1), and in which interference is allowed to affect only the trace involved in intrusions. There are various accounts of this type in the clinical literature (for a review see Dalgleish, 2004), but the most prominent one is dual representation accounts (Bisby & Burgess, 2017; Brewin, 2014; Brewin et al., 1996, 2010). Such accounts suggest that intrusive reexperiencing and voluntary retrieval of trauma are governed by distinct memory systems, with intrusions supported by a specialized long-term perceptual memory system that is functionally dissociable from the episodic memory system supporting voluntary recall of the same event (Brewin, 2014). The former system is thought to be preferentially susceptible to our sensory-perceptual/visuospatial (Tetris) interference task (Brewin, 2014; Brewin et al., 1996; Holmes et al., 2004), consistent with our findings.

Our result that the interference effect on intrusions did not appear to arise from changes in perceptual priming appears at odds with clinical accounts of intrusive symptom development in PTSD (Brewin, 2014; Ehlers & Clark, 2000; Holz et al., 2014; Michael & Ehlers, 2007; Sündermann et al., 2013), although intrusions and priming could still be linked through other means. Our intrusion/priming dissociation is more compatible with the widely accepted distinction between nondeclarative (supporting priming) and declarative memory systems (supporting intrusions; Berntsen, 2009). In other words, what seems to distinguish intrusive memories is the *conscious* involuntary retrieval, unlike implicit priming which is thought to involve *unconscious* involuntary retrieval; Berntsen, 1996).

Consolidation is assumed to be a slow and time-dependent memory process, hence influences on it may become apparent only after a delay (e.g., after hours/days or after sleep) but not necessarily sooner (Dudai, 2004; McGaugh, 2000, 2015; Nader, 2003). Our interference effects on intrusions were almost immediate, casting doubt on whether such effects arise from changes in consolidation as previously assumed (Deeprose et al., 2012; Holmes et al., 2009; Holmes, James, et al., 2010). It is also possible that effects on early intrusions (e.g., attributable to temporary interference) differ from those on later intrusions (e.g., attributable to consolidation). Nevertheless, such assumptions on the time course of (emotional) memory consolidation currently rely on rodent studies and using paradigms that tap into nondeclarative memory, including fear conditioning and instrumental learning (McGaugh, 2015; Miserendino, Sananes, Melia, & Davis, 1990; Nader, 2003; Schafe & LeDoux, 2000; Visser et al., 2018). In contrast, the same assumptions are not fully endorsed in human studies using paradigms that tap into declarative memory (Dewar, Cowan, & Sala, 2007; Wixted, 2004), which we assume support conscious aspects of intrusions. It therefore currently remains unclear when consolidation begins or ends for human declarative memories, leaving open the possibility that our effects are still related to consolidation.

##### Methodological Considerations

One consideration is whether procedures used with the trauma film paradigm (James, Lau-Zhu, Clark, et al., 2016; Lau-Zhu et al., 2018) extend to real-life trauma and clinical populations. Indeed, our interference procedure (initially developed in the laboratory) has recently been shown to reduce intrusive memories after real-life trauma (Horsch et al., 2017; Iyadurai, Blackwell, et al., 2018; Kessler et al., 2018) albeit in early and proof-of-concept stage findings warranting further enquiry. Diagnostic criteria for PTSD now allow indirect exposure to trauma via film footage to fulfill criteria for trauma exposure (so long as it is work-related), for instance, journalists who perform news editing (APA, 2013). There is also increased recognition that exposure to traumatic events via electronic mediums (e.g., film footage) can also result in stress-related symptoms that warrant further scrutiny (Holman, Garfin, & Silver, 2014; Silver et al., 2013).

Another potential criticism relates to the use of a diary to record intrusive memories in daily life, where the conditions that elicit intrusions (e.g., retrieval cues) are difficult to control for. However, our findings on intrusions converged across assessments, both in the diary and in the laboratory (with presumably higher level of experimental control). One may also argue that self-report such as for reporting intrusions is subjected to demand characteristics, yet our findings suggest that groups were matched on expectations about the direction of the interference effects (see the online supplemental materials), and demand ratings are typically ruled out as a confound in trauma film studies (James, Lau-Zhu, Clark, et al., 2016; Lau-Zhu et al., 2018). Future research should continue to leverage laboratory assessments of intrusions informed by a modeling of factors that govern everyday intrusions (Lau-Zhu et al., 2018; Takarangi et al., 2014), as well as assess other concomitant affective outcomes such as physiological correlates (Kunze, Arntz, & Kindt, 2015; Visser et al., 2018; Wegerer, Blechert, Kerschbaum, & Wilhelm, 2013).

The absence of interference on some of the memory tasks (i.e., those not assessing intrusions) could reflect lack of statistical power (Anderson, Kelley, & Maxwell, 2017), as we mainly powered our study on the basis of effect sizes for intrusion effects. Nevertheless, the interference effects in free recall and priming (Experiment 1) and in attentional bias (Experiment 2) were numerically in the opposite direction to that in intrusions, and thus it does not appear to be the case that a trend just failed to reach significance because of low power. This interpretation was further corroborated by additional ANOVAs on standardized scores—which demonstrated the effect sizes for intrusions were significantly bigger than in the other measures (this interaction would be unlikely to be significant if the other measures were just extremely noisy)—as well as additional analyses using an Bayesian approach supporting the relevant lack of group differences (see the online supplemental materials).

Further converging evidence with our current memory dissociation findings could be sought in at least three ways. First, more stringent between-groups designs could be used—where each participant is given only a single retrieval task—to fully rule out contamination effects across memory tasks that could potentially arise from the fixed-order designs used in our three experiments. Second, additional task comparisons could account for other differences between measures of intrusive/involuntary and voluntary memories not directly addressed here, such as the use of frequency versus accuracy as main outcomes. Although there was a strong correspondence between frequency count and accuracy within the diary (proportions of reported intrusions matched with film scenes were 87% to 98%), additional evidence they are assessing a similar construct should be explored. Other retrieval factors to account for include the requirement for monitoring (Vannucci et al., 2014), the ease of retrieval (Barzykowski & Staugaard, 2016; Uzer, Lee, & Brown, 2012), and types of triggers (Berntsen, 2009; Berntsen et al., 2013; Mace, 2004; Staugaard & Berntsen, 2014). Third, there remains the possibility that each measure may not be pure, mixing involuntary and voluntary contributions (Hellawell & Brewin, 2002; Mace, 2014; Richardson-Klavehn & Bjork, 1988; Whalley et al., 2013). Alternative approaches can be considered to dissociate controlled from automatic contributions within a given task (Yonelinas & Jacoby, 2012).

Our selective interference effects remain to be demonstrated with other memory paradigms. Although the impact of postencoding interference on subsequent memory has been demonstrated using a variety of episodic materials (other than trauma films), such studies tend to use nonemotional stimuli (e.g., objects; Hupbach, Gomez, Hardt, & Nadel, 2007; Hupbach, Gomez, & Nadel, 2009), focus on voluntary retrieval (Chan & LaPaglia, 2013; Schwabe & Wolf, 2009; Wichert, Wolf, & Schwabe, 2013), or consider other forms of clinically relevant outcomes, such as ratings of vividness/emotionality (Engelhard et al., 2010; Leer et al., 2017; Tadmor, McNally, & Engelhard, 2016; van den Hout et al., 2014). Some of these have also found that reductions in vividness/emotionality (of nonaversive stimuli) were accompanied by worsening of recognition performance (Leer et al., 2017; van den Hout, Bartelski, & Engelhard, 2013), suggesting that not all interference effects are selective, unlike in our experiments. Nevertheless, it is difficult to draw direct comparisons, as *involuntary* retrieval (a key feature of intrusive memory) is not directly addressed in such studies. It would be of great interest for future research to combine these various lines of investigation of the effects of postencoding interference on different stimuli/measures.

### Conclusions and Future Directions

Our results of a selective interference effect on intrusive memories highlight the need for theories of episodic memory to accommodate findings on intrusive/involuntary forms of memories, and to extend clinical theories such as dual representation accounts. They may also inform clinical interventions seeking to selectively target intrusive memories without erasing voluntary memories of emotional events. Future research should further dissect mechanisms underlying the effects discussed. These include the timing of the intervention in relation to film viewing (James, Lau-Zhu, Tickle, et al., 2016), the specificity as well as timing of delivery of the reminder cue (Horsch et al., 2017; Iyadurai, Blackwell, et al., 2018; James et al., 2015), the nature of the event (Arnaudova & Hagenaars, 2017; Davies et al., 2012; Lang et al., 2009), and aspects related to the interference task, to resolve controversies around issues of task modality (Hagenaars et al., 2017; Holmes, James, et al., 2010; Lau-Zhu et al., 2017) and individual task performance (James et al., 2015; Lau-Zhu et al., 2017). Another important issue that merits further investigation is how intrusive memories are experienced once they emerge (Lau-Zhu et al., 2018; Marks, Franklin, & Zoellner, 2018) and how they may impact an individual’s daily functioning (Iyadurai, Visser, et al., 2018). We hope such fine-grained investigations will further constrain theories on intrusive memories and their relationship to voluntary memory of emotional events, and help optimize translational parameters.

### Context Paragraph

This series of experiments tackled one of the most heated debates in the literature on intrusive memories (single vs. separate-trace accounts). We began a research program involving clinical and basic memory researchers to help resolve this long-standing controversy in the trauma-film literature spanning the last two decades. This collaboration showcases the benefits of taking an experimental approach to study psychopathology, in terms of advancing cognitive theories, and in doing so, promoting clinical innovations. The interference procedure used has already shown initial early stage promise to prevent intrusive memories of real-life traumas (Horsch et al., 2017; Iyadurai, Blackwell, et al., 2018). Experimental studies can further illuminate the theoretical basis of such therapeutic gains to refine translational parameters. An exciting opportunity is to extend novel applications for clinical areas beyond trauma where intrusive imagery is increasingly recognized as a promising intervention target. These areas include hypomania (Davies et al., 2012), affect lability (Di Simplicio et al., 2016), visceral syndromes (Kamboj et al., 2015), cravings (Skorka-Brown, Andrade, Whalley, & May, 2015), as well as ubiquitous yet unaddressed anxiety across typical and atypical development (Burnett Heyes, Lau, & Holmes, 2013; Ozsivadjian, Hollocks, Southcott, Absoud, & Holmes, 2017).

## Supplementary Material

Supplementary material

## Figures and Tables

**Table 1:** Means and Standard Deviations by Group for Outcomes in Measures of Memory of the Trauma Film in Experiment 1

Measure	Reminder-plus-Tetris (*n* = 23) *M* (*SD*)	Reminder-only (*n* = 23) *M* (*SD*)
Intrusion diary		
Number of intrusions over one week^a^	2.70 (2.53)	5.61 (3.41)
Recognition task		
Hits	69.17 (9.79)	70.83 (7.66)
FA	24.17 (14.27)	25.43 (8.18)
Priming task		
Film trials RT (sec)	4.14 (0.74)	3.98 (0.77)
Foil trials RT (sec)	4.28 (0.77)	4.08 (0.77)
Recall task		
FR: Event details	57.91 (29.24)	50.39 (24.98)
FR: Perceptual details	7.91 (6.40)	8.96 (8.88)
SP: Event details	96.78 (34.78)	94.30 (35.62)
SP: Perceptual details	20.04 (14.96)	24.70 (17.07)

*Note*. FA = false alarm; RT = reaction times; FR = free recall; SP = specific probing.

^a^This is also reported in Figure 4, but repeated here for comparability across the four memory measures.

**Table 2:** Means and Standard Deviations by Group for Outcomes in Measures of Memory of the Trauma Film in Experiment 2

Measure	Reminder-plus-Tetris (*n* = 18) *M* (*SD*)	Reminder-only (*n* = 18) *M* (*SD*)
Intrusion diary (Days 1 to 7)		
Number of intrusions over one week	2.50 (2.53)	8.28 (6.15)
Vigilance-intrusion tasks		
Number of early intrusions (Day 1)	7.22 (4.56)	13.28 (7.70)
Number of later intrusions (Day 8)	5.00 (6.36)	9.28 (3.95)
Recognition task (Day 8)		
Hits	56.39 (12.93)	54.67 (16.61)
FA	15.22 (11.23)	19.72 (14.15)
Attentional-capture task (Day 1)		
Accuracy	0.98 (0.02)	0.97 (0.07)
Emotional stills (sec)	0.011* (0.019)	0.008* (0.018)
Neutral stills (sec)	–0.002 (0.024)	0.002 (0.023)

* Significant one-sample *t* tests (one-tailed; *p* < .10), meaning that a bias score was above chance—mainly attributable to trauma film stills with emotional content.
