# Supplementary material for: Intrusive Memories and Voluntary Memory of a Trauma Film: Differential Effects of a Cognitive Interference Task After Encoding
Source: J Exp Psychol Gen. Author manuscript; Available in PMC 2020 Dec 21. (PMC7116494; doi:10.1037/xge0000598)
Supplement: Supplementary material [file EMS107276-supplement-Supplementary_material.PDF]

Supplemental Materials

Lau-Zhu, A., Henson, R.N., & Holmes, E.A. Intrusive Memories and Voluntary Memory of a Trauma Film: Differential Effects of a Cognitive Interference Task After Encoding.

Experiment 1

Additional Methods.

Trauma film. Multiple clips of scenes with different content are often used in trauma film research rather than one long clip (James, Lau-Zhu, Clark, et al., 2016). The rationale to have variety in content is so that the paradigm produces sufficient intrusions across participants. The specific scene for which an individual will have an intrusive memory is idiosyncratic, and typically if we show 11 different scenes then participants may on average have, for example, three different scenes that intrude (but which of the 11 scenes intrude varies between participants, and each scene may intrude more than once) (Bourne, Mackay, & Holmes, 2013; Clark, Holmes, Woolrich, & Mackay, 2016). For example, one participant may develop three intrusions: the body of a dead child being dragged on the street, a red car hitting a wall, a scalpel during surgery; but different participant may have 3 intrusions but to other clips that were shown. This number of intrusions with different content is similar to the number of intrusive "hotspots" in studies of intrusive memories of patients with post-traumatic stress disorder (PTSD) (Grey & Holmes, 2008; Holmes, Grey, & Young, 2005) – that is the number of different worst moments within a trauma that reoccur as intrusive memories.

Filler tasks. For the knowledge search task (10 min each time), participants were presented with a list of questions which they had to search the answer for using the encyclopaedia *Enquire Within Upon Everything* (Bremner, 1988) in book form. For the music

filler task (10 min), participants listened to classical music excerpts and rated them on a Likert-scale from 1 ‘not at all pleasant’ to 9 ‘extremely pleasant’ (Holmes, James, Coode-Bate, & Deeprose, 2009; Holmes, James, Kilford, & Deeprose, 2010; James et al., 2015). The music filler task was programmed using E-Prime 2.0 (Schneider, Eschman, & Zuccolotto, 2002) and played via headphones.

Self-report measures. Baseline depressive symptoms were assessed using the Beck Depression Inventory Second Edition (BDI-II; Beck, Steer, & Brown, 1996). The BDI-II consists of 21 items (each measured on a scale from 0-3) and shows good internal validity (alpha = .81; Beck, Steer, & Garbin, 1988). Trait anxiety was assessed using the State-Trait Anxiety Inventory – Trait Version (Spielberger, Gorsuch, Lushene, Vagg, & Jacobs, 1983). The STAI-T consists of 20 items (on a scale from 1-4) and shows good internal validity (alpha = .90; Spielberger, Reheiser, Owen, & Sydeman, 2004). Prior trauma history was assessed using the Traumatic Experience Questionnaire (TEQ), which is a 12-item checklist adapted from the Criterion A list of the Posttraumatic Diagnostic Scale (Foa, 1995). General use of mental imagery in everyday life was assessed using the Spontaneous Use of Imagery (SUIS; Reisberg, Pearson, & Kosslyn, 2003). The SUIS consists of 12 items (each measured on a scale from 1-5) and shows excellent internal consistency (alpha = .98; Reisberg et al., 2003).

Three visual analogue scales (VAS) were used to assess *negative mood* states (sadness, depression, and hopelessness) and were anchored from 0 (not at all) to 10 (extremely). Participants were instructed to rate each word according to their feelings ‘at the moment’. A composite score was obtained by summing up across mood states (James et al., 2015; James, Lau-Zhu, Tickle, Horsch, & Holmes, 2016).

The amount of *attention* paid to the film (attention to film) and the *personal relevance* of the film (film relevance) were both assessed using 11-point scales ranging from 0 (not at all) to 10 (extremely).Compliance with completing the diary (*diary compliance*) was assessed using a single VAS, which was anchored from 0 (not at all accurate) to 10 (extremely accurate).

Expectation regarding the task manipulation (*demand rating*) was assessed using a 21-point scale in response to the question '*How much do you predict that performing the Tetris task after a distressing film (rather than watching it normally) would increase or decrease intrusive images of the film of the type you recorded in your diary*?'. This scale was anchored from -10 (extreme decrease), to 0 (no impact), to 10 (extreme increase).

Free-recall task. Pilot work on this task with three volunteers with open feedback was used to determine parameters such as typical recall duration, ease of understanding of the instructions, appropriateness of the stimuli, and ease of using the recorder. For specific probing, at the end of each of the 11 cues they would hear a beep sound signalling that the time limit was reached and the next cue phrase appeared on screen. Here they were specifically encouraged to retrieve as many perceptual details (e.g., objects, colours and sounds) as they could. In line with the Autobiographical Interview (Levine, Svoboda, Hay, Winocur, & Moscovitch, 2002; McKinnon et al., 2014), event details referred to what had happened, such as the people that were present, their behaviours and their actions; these also included emotions and thoughts of the people involved in the film. Perceptual details referred to information experienced through different sensory modalities, and for the trauma film these would include visual (e.g., objects and colours) and auditory details (e.g., sounds). When a detail could be scored as either event or perceptual detail, preference was given to the more specific category (i.e., perceptual).

Priming task. Foil stills were obtained from a variety of sources, including footage of other parts of the same films that had been edited out (thus not shown as part of the trauma film in the experiment), footage from other films, and stills from other online sources. An independent norming study indicated that the film and foil sets were matched on various emotionality indices. Any content from a single full still was presented once only. In a given trial, the left still-half was displayed on the right side of the screen and the right still-half was displayed on the left. That way, if both halves did happen to come from the same original still – that is, if they did indeed match – it would be much more difficult for the participant to identify this simply by noticing contiguities across the halves' adjoining inner edges, because these inner edges would not appear next to each other on the screen. We reasoned that participants would thus be more likely to rely on their memory of the film still rather than on features of the stills themselves. Participants were given a practice with 12 trials with a separate set of foil stills not presented in the main priming task.

Additional Results.

Baseline measures, mood and task manipulation checks. Ethnicities were described by participants as: 33% White British, 24% White Other, 17% Asian (Indian, Pakistani or other), 15% Chinese, 9% Mixed and 2% Black Caribbean. Groups did not significantly differ at baseline on gender, χ^2 (1, N = 46) = 0.37, p = .763, age, depressive symptoms, trait anxiety, the number of previous traumatic events or their general use of imagery, t's < 1. Viewing the film resulted in predicted increases in negative mood, F(1,44) = 46.24, p < .001, η_p^2 = .512, but there were no group differences in overall negative mood or in degree of mood drop, F's < 1. There were also no significant group differences in ratings for attention to film, personal relevance of the film, demand ratings or diary compliance, t's < 1 (see Table S1 for relevant descriptive statistics).

[Insert Table S1]

Intrusion diary. The rating of diary compliance suggested a good level of self-reported accuracy (Table S1), also in line with previous studies (Deeprose, Zhang, Dejong, Dalgleish, & Holmes, 2012; Holmes et al., 2009; James, Lau-Zhu, Tickle, et al., 2016). Examples of intrusive memories as written in the diaries are 'man flailing', 'the man with the razor cutting himself', and 'I saw an image of the eye'. In their diaries, participants reported an associated identifiable cue for the majority of their intrusions, both overall (80.1%) and within each group (reminder-plus-Tetris: 79.0%; reminder-only: 80.6%). Examples of the reported cues are 'saw a clip of the ocean', 'shave in the morning' and 'talking about optometry with a friend'. Informed by a classification system (Mace, 2004), we further classified cues as either *external* (experienced in the environment, including all sensory and perceptual experiences, including activities) or *internal* (those with internal source only, such as bodily sensations or states, emotional states, and thoughts). Among the reported cues, the majority were classifiable as external, both overall (88.9%) and within each group (reminder-plus-Tetris: 91.8%; reminder-only: 87.5%), and almost none were classifiable as internal, both overall (2.0%), and within each group (reminder-plus-Tetris: 0.0%, reminder-only: 2.9%). The rest were not classifiable as either external or internal. The number of intrusions on the one-week diary did not significantly correlate with i) any of the baseline self-report measures (BDI-II, STAI-T, TEQ, SUIS), or ii) changes in negative mood from before to after watching the film, either overall (r's = -0.22-0.23, p's > .288), or within each group separately (r's = -0.19-0.34, p's > .108).

Recognition task. High-confidence responses are more likely to be associated with recollection than low-confidence responses (Yonelinas, 2002), thus a selective interference on 'recollection' would predict significant group differences on recognition within high-

confidence responses but not (or at least to a smaller extent) within low-confidence responses. We ran a 2 (within-group: high vs. low-confidence) × 2 (between-group: reminder-only vs. reminder-plus-Tetris) mixed ANOVA on recognition scores. As expected, these analyses revealed that recognition accuracy was higher within high-confidence responses (scores of 3-4; $M = 0.54$, $SE = 0.03$) than low-confidence responses (scores of 1-2; $M = 0.17$, $SE = 0.03$), $F(1,32) = 97.80$, $p < .001$. However, the main effect of group was not significant, nor the group × confidence interaction (F's <1). The same pattern of findings remained when high-confidence responses were restricted to confidence scores of 4 only. Overall, these results support the absence of group differences on recognition accuracy across both confidence types, and therefore the lack of a selective interference on 'recollection'.

Comparing retrieval intention and cue overlap. An important distinction between the diary and the other measures is that the diary recorded intrusions on Days 1 to 7 consecutively whereas the other measures were delivered on Day 8. To control for such differences in post-encoding delay, we repeated the analyses above by restricting the number of diary intrusions to the final day available in the diary, that is, Day 7 (reminder-plus-Tetris: $M = 0.09$, $SD = 0.29$; reminder-only, $M= 0.48$, $SD = 0.73$) so that this was better matched to the post-encoding delay of the other memory measures (Day 8). The same pattern of results emerged (i.e., showing that there is a selective interference effect on diary intrusions but not on the other memory measures), although now nonparametric tests showed that the critical three-way interaction between group × intention × cues only showed a non-significant trend, $U(44) = 182.00$, $Z = 1.81$, $p = .070$.

Bayesian analyses. For our three key non-significant results in recognition, priming and free recall, exploratory Bayesian analyses were conducted to help assess the relative data likelihood under the null versus the alternative hypothesis, using Gönen's method (Gönen,

Johnson, Lu, & Westfall, 2005). We used the effect size of the interference effect on diary intrusions as the prior (d = .97). A Bayes factor above 3 can be interpreted as evidence supporting the null. The associated Bayes factors were BF_{01} = 18.97, 11.49 and 13.20, for recognition, priming, and recall, respectively, thus suggesting that the data were strongly more likely under the null hypothesis (i.e., no effect of interference on these tasks).

Experiment 2

Additional Methods.

Recognition task. This was the same as in Experiment 1, except that participants had 5 sec to make a remember/know (R/K) judgement after each 'yes' response (instead of providing confidence ratings after all responses). R judgments referred to instances when recognition was accompanied by a conscious sense of *recollection*, whereby some other aspects of what happened in the film came to awareness; K judgments referred to instances when one was certain that they recognised the picture (*familiarity*) but nevertheless did not consciously recollect anything else about the film (Rajaram, 1993).

Vigilance-intrusion task. The digits used as part of the vigilance task (i.e., presented on top of either the black background of blurred film/foil scenes; see Main Manuscript) were white and chosen randomly from five different font sizes (48, 72, 94, 100 and 120 points; Arial font type) corresponding to stimulus heights of 12-29 mm approx. Each foil still appeared on one trial only, whereas each film still appeared on two trials. Film stills were never on a same trial as the target digit '3'. Prior to the main vigilance-intrusion task, participants completed 36 practice trials to familiarise themselves with the digit-vigilance component of the task.

Attentional-capture task. This location of the target (one or two dots) was determined by randomly selecting a point within an imaginary 2 cm × 2 cm square behind the centre of the film/foil still on either the left or right side of the screen. The precise location of the target was randomly determined to fall within this square. Participants were instructed to identify whether the target had either one or dot dots, and had the opportunity of a short break between each of the four runs of this task. Twelve practice trials with a different set of foil stills were given prior the four experimental runs.

Additional Results.

Baseline measures, mood and task manipulation checks. Ethnicities were described by participants as: 42% White Other, 28% White British, 14% Asian (Indian, Pakistani or other), 8% Chinese, 3% Mixed, 3% Black African, and 3% as any other. Groups were not significantly different at baseline in terms of gender, χ^2 (1, N = 36) = 0.11, p = .738, age, depressive symptoms, trait anxiety, the number of previous traumatic events and their general use of imagery, t's < 1.35. Viewing the film resulted in predicted increases in negative mood, $F(1,34) = 48.18$, $p < .001$, $\eta_p^2 = .586$, and there were no significant group differences in the overall negative mood, $F(1,34) = 4.01$, $p = .053$, or in mood drop, $F < 1$. There were also no significant group differences in ratings for attention to film, personal relevance of the film, and demand ratings, t's < 1. The reminder-plus-Tetris group reported higher ratings for diary compliance than the reminder-only group, $t(34) = 2.46$, $p = .019$, $d = .81$. See Table S2 for descriptive statistics.

[Insert Table S2]

Intrusion diary. The rating of diary compliance suggested a good level of self-reported accuracy (Table S2), also in line with previous studies (Deeprose et al., 2012;

Holmes et al., 2009; James et al., 2015). Examples of intrusive memories as written in the diaries are ‘I saw kid by road side’, ‘legs crushed against a stone wall’, and ‘program in Rwanda’. Similar to Experiment 1, participants reported in their diaries that the majority of their intrusive memories were associated with a cue, both overall (70.3%) and within each group (reminder-plus-Tetris: 67.9%; reminder-only: 71.1%). Examples of the reported cues are: ‘many kids crossing the road’, ‘passing by a field with horses’ and ‘my landlord talking about ISIS’. Using the same criteria as in Experiment 1, among the reported cues, the majority were classifiable as *external*, both overall (79.6%) and within each group (reminder-plus-Tetris: 83.3%; reminder-only: 78.3%), and only a minority were classifiable as *internal*, both overall (9.9%), and within each group (reminder-plus-Tetris: 13.9%, reminder-only: 8.5%). The rest were not classifiable as either external or internal. The number of intrusions on the one-week diary did not significantly correlate with i) any of the baseline questionnaires (BDI-II, STAI-T, TEQ, SUIS) or ii) changes in negative mood before to after watching the film, either overall (r’s = -0.13-0.30, p’s > .080), or within each group separately (r’s = -0.21-0.36, p’s > .141).

Recognition task (Day 8). R/K responses were not collected for two participants from the reminder-only group due to error with the software. Around 65% of hits were accompanied by R responses. With the available data, recognition accuracy scores were calculated separately for trials endorsing R vs. K judgments. A 2 (between-group: reminder-plus-Tetris vs. reminder-only) × 2 (within-subject: R vs. K) mixed ANOVA revealed a significant judgement effect, $F(1, 32) = 30.09$, $p < .001$, $\eta p^2 = .485$, suggesting that recognition accuracy was higher for trials endorsing R judgements (M = 0.34, SE = 0.02) than for trials endorsing K judgements (M = 0.11, SE = 0.02). There were no main effect of group nor significant group × judgment interaction, F’s < 1. These findings indicate an absence of group differences regardless of R/K judgements.

Vigilance-intrusion task (Day 8). In the control condition (reminder-only group), the mean number of intrusions (range = 0 to 17; Table 2) appeared to be higher than that reported in the control condition in the study by James et al. (2015; mean of 3-4 intrusions), where a different and shorter (2-min) laboratory test was used (intrusion provocation task). Note that on few occasions, some participants provided more than one description per key press if they happened to have intrusions of more than one film clip. There was a significant positive correlation between the number of intrusion descriptions provided and the number of intrusion key-presses, $r = 0.99$, $N = 36$, $p < .001$, suggesting that participants indeed used the key presses to index intrusions of the film.

The number of intrusions on the vigilance-intrusion task delivered on *Day 8* did not significantly correlate with any of the baseline questionnaires BDI-II, STAI-T, TEQ, SUIS) either overall (r's = 0.00-0.18, p's > .294) or within each group separately (r's = -0.04-0.32, p's > .203). The number of intrusions on the same task did significantly and positively correlated with changes in negative mood from before to after watching the film within reminder-only group only ($r = 0.62$, $p = .006$), as confirmed by Fisher's tests ($p = .029$), thus suggesting that in this group more mood drop after the film was associated with a higher number of intrusions. However, adding mood drop as a covariate in the relevant analyses did not change the pattern of results.

We ran exploratory analyses to seek for further evidence that participants reported intrusive memories following exposure to the blurred film stills within the vigilance-intrusion task. For each participant, we computed the proportion of trials with film stills and of trials with foil stills that were subsequently followed by an intrusion key-press within three trials from still presentation. We first report the relevant data on the vigilance-intrusion task completed on Day 8. A 2 (between-group: reminder-plus-Tetris vs. reminder-only) × 2

(within-group: film vs. foil trials) mixed model ANOVA revealed a main effect of trial, $F(1,34) = 34.43$, $p < .001$, $\eta_p^2 = .503$, suggesting that participants were indeed more likely to indicate experiencing an intrusion after encountering a film still ($M = 0.24$, $SD = 0.23$) than a foil still ($M = 0.03$, $SD = 0.02$). The main effect of group was also significant, $F(1,34) = 4.43$, $p = .043$, but not the group × trial interaction, $F(1,34) = 2.32$, $p = .137$.

Vigilance-intrusion task (Day 1). There was a significant positive correlation between the number of intrusion descriptions and the number of intrusion key-presses, $r = 0.88$, $N = 36$, $p < .001$. This suggests that intrusion key-presses are reliable indicators for intrusions of the film. The number of intrusions on the vigilance-intrusion tasks delivered on *Day 1* did not significantly correlate with i) any of the baseline questionnaires (BDI-II, STAI-T, TEQ, SUIS) or ii) changes in negative mood before to after watching the film, either overall (r's = -0.11-0.24, p's > .167) or within each group separately (r's = -0.42-0.35, p's > .079).

We ran exploratory analyses to seek for further evidence that participants reported intrusive memories following exposure to the blurred film stills within this task on *Day 1*, similar to findings on *Day 8.* A 2 (between-group: reminder-plus-Tetris vs. reminder-only) × 2 (within-group: film vs. foil trials) mixed model ANOVA revealed that the main effect of group was not significant, $F(1,34) = 3.17$, $p = .084$, nor the group × trial interaction, $F < 1$. However, there was a significant main effect of trial, $F(1,34) = 41.40$, $p < .001$, $\eta_p^2 = .549$, again suggesting that participants were indeed more likely to indicate experiencing an intrusion after encountering a film still ($M = 0.32$, $SD = 0.26$) than a foil still ($M = 0.15$, $SD = 0.12$).

A multiple regression model was used to investigate whether the number of early laboratory-intrusions (in the vigilance-intrusion task on Day 1) predicted subsequent diary intrusions summed over the following week. The main predictors (laboratory intrusions and

group) were entered into a first block and the interaction term in a second block. The model with both predictors was significant, $F(2,33) = 13.54$, $p < .001$, $R^2 = .45$, confirming that a higher number of diary intrusions was associated with a higher number of early laboratory-intrusions, b = 0.36, $SE_b = 0.11$, $\beta = .45$, $p = .003$, and with being in the reminder-only group, b = -3.61, $SE_b = 1.56$, $\beta = -.33$, $p = .027$. The model with the interaction term did not result in a significant R^2 change, $F < 1$. Thus, critically the number of early intrusions as assessed in a 9-min task soon after interference within the laboratory was predictive of later intrusions in a one-week diary in daily life, irrespective of group allocation.

We then compared the size of the effect on the vigilance-intrusion task on Day 1 versus Day 8. A 2 (between-group: reminder-plus-Tetris vs. reminder-only) × 2 (within-group: Day 1 vs. Day 8) mixed model ANOVA revealed an expected main effect of group, $F(1,34) = 9.06$, $p = .005$, $\eta_p^2 = .210$, showing that overall the reminder-plus-Tetris group ($M = 6.11$, $SE = 1.21$) reported fewer intrusions than the reminder-only group ($M = 11.28$, $SE = 1.21$). Further, there was a main effect of delay interval, $F(1,34) = 11.60$, $p = .002$, $\eta_p^2 = .254$, with more intrusive memories being reported on Day 1 ($M = 10.25$, $SE = 1.05$) compared to Day 8 ($M = 7.14$, $SE = 0.88$). The interaction between group and delay interval was not significant, $F < 1$, crucially suggesting that the interference effect was revealed irrespective of the delay interval. Finally, test-retest reliability between the number of intrusions on the vigilance-intrusion tasks on Day 1 and Day 8 was good, either overall (Cronbach's alpha = 0.77) or within each group (reminder-plus-Tetris = 0.70; reminder-only: 0.73), suggesting that a consistent number of intrusions were reported between both time points.

Attentional-capture task (Day 1). Analyses were collapsed across runs and the lags between still and target probe (500 and 1000 msec) because preliminary analyses showed no evidence that these interacted with the other variables of interest. We ran a 2 (between-group:

reminder-plus-Tetris vs. reminder-only) × 2 (within-group: emotional vs. neutral still pairs) mixed ANOVA on attentional bias scores. This confirmed a significant main effect of emotionality, $F(1,34) = 4.12$, $p = .050$, $\eta_p^2 = .108$. Neither the main effect of group nor interaction between group × emotionality was significant, *F's* < 1. Taken together, these findings suggest that while an attentional capture to trauma film cues was detectable when those stills depicted emotional scenes, the degree of such bias appeared to be equivalent between the reminder-only and the reminder-plus-Tetris groups.

We also ran a series of correlational analyses to explore whether attentional bias scores were related to intrusion rates (i.e., in the diary and the vigilance-intrusion tasks). Attentional bias to trauma-film stills were not significantly correlated with any intrusion measure, either overall (*r*'s = -.17 to .05, *p's* > .32) or within each group separately (*r*'s = -.55 to .05, *p's* > .07). Attentional bias to emotional-stills only were also not significantly correlated with intrusion measures, either overall (*r*'s = -.26 to .05, *p's* > .13) or in each group (*r*'s = -.18 to .14, *p's* > .47). The only exception was the significant correlation between attentional bias to emotional-stills and the number of diary intrusions in the reminder-plus-Tetris group ($r = -.63$, $p = .006$). However, this was in the opposite direction of what would have been predicted (i.e., more bias associated with fewer intrusions), and would not remain significant after controlling for multiple comparisons.

Bayesian analyses. For our two key non-significant results in recognition and attentional capture, exploratory Bayesian analyses were also conducted to help assess the relative evidence for the null versus the alternative hypothesis, similar to Experiment 1. For recognition, we used effect size of the interference effect on vigilance-intrusion task on the same day (Day 8) as the prior ($d = 0.81$), revealing a Bayes factor of $BF_{01} = 12.72$. For attentional capture (attentional bias to emotional film-stills), we used the effect size of the

interference effect on vigilance-intrusion task on the same day (Day 1) as the prior ($d = 0.92$), revealing a Bayes factor of $BF_{01} = 49.33$. Results on both analyses suggest that the data were strongly more likely under the null (i.e., no interference effect on a particular measure).

Experiment 3

Additional Methods.

Vigilance-intrusion task with estimates. If at least one intrusion occurred on each 3-min run, participant then completed three visual analogue scales (VAS) ranging from 0 (not at all) to 100 (extremely) in relation to those intrusions for the overall 3-min period. These scales assessed distress (*how distressing did you find these image-based memories?*), vividness (*how vivid did you find these image-based memories?*) and sense of 'nowness' (*to what extent did you feel you were watching the film again when these image-based memories popped up?*). Available ratings were averaged across the three 3-min runs within each retrieval load condition.

Additional Results.

Baseline measures, mood and task manipulation checks. Ethnicities were described by participants as: 53% White British, 21% White Other, 12% Asian (Indian or Pakistani), 5% Chinese, 5% Mixed, and 4% Black Caribbean. Groups were not significantly different at baseline on gender, $\chi^2(1, N = 57) = 1.02, p = .60$, age, depressive symptoms, trait anxiety, the number of previous traumatic events, or their general use of imagery, F's < 2.59. Viewing the film resulted in predicted increases in negative mood, $F(2,54) = 49.92, p < .001, \eta_p^2 = .480$, and there were no significant group differences in the overall negative mood or in mood drop, F's < 1. There were also no significant group differences on ratings for attention to film,

personal relevance of the film and demand ratings, *F*'s < 1.32. See Table S3 for descriptive statistics.

[Insert Table S3]

Vigilance-intrusion task with key presses. All the following analyses were restricted to the two main groups of interest: reminder-plus-Tetris and reminder-only. The number of intrusions on the vigilance-intrusion task with *key presses* did not significantly correlate with i) any of the baseline questionnaires (BDI-II, STAI-T, TEQ, SUIS) or ii) changes in negative mood before to after watching the film, either overall (*r*'s = 0.05-0.22, *p*'s > .098) or within each group separately (*r*'s = -0.15-0.34, *p*'s > .156).

We also ran exploratory analyses to seek for evidence that participants reported intrusive memories following exposure to the blurred film stills within this task, following the same procedure as in Experiment 2. A 2 (between-group: reminder-plus-Tetris and reminder-only) × 2 (within-group: film and foil trials) mixed model ANOVA revealed a main effect of trial, $F(1,36) = 58.49$, $p < .001$, $\eta_p^2 = .619$, critically suggesting that participants were indeed more likely to indicate experiencing an intrusion after presentation of a film still (*M* = 0.37, *SD* = 0.26) rather than a foil still (*M* = 0.09, *SD* = 0.09), in line with findings from Experiment 2. There was also a main effect of group, $F(1,34) = 9.34$, $p = .004$. The group × trial interaction was not significant, $F < 1$.

Vigilance-intrusion task with estimates. All the following analyses were restricted to the two main groups of interest: reminder-plus-Tetris and reminder-only. For the finger tapping task, there was no significant group differences in the number of key presses per min (reminder-only: *M* = 68.74, *SD* = 30.12; reminder-plus-Tetris: *M* = 58.80, *SD* = 28.06), or the percentage of total correct 5-key sequences (reminder-only: *M* = 74.67, *SD* = 22.47;

reminder-plus-Tetris: $M = 71.49$, $SD = 24.28$) during the tapping, t's < 1.05, suggesting similar success in performing the finger tapping task.

For the counting backwards task, the two groups also did not significantly differ in the total numbers counted per min (reminder-only: $M = 29.63$, $SD = 6.95$; reminder-plus-Tetris: $M = 30.31$, $SD = 7.23$) or the percentage of total correct numbers (reminder-only: $M = 97.76$, $SD = 2.25$; reminder-plus-Tetris: $M = 96.79$, $SD = 2.94$) during the counting, t's < 1.14, suggesting similar success in performing the counting backwards task.

The number of intrusions on the vigilance-intrusion tasks with *estimates* (in each of the three retrieval load conditions) did not significantly correlate with i) the BDI-II, STAI-T or ii) changes in negative mood before to after watching the film, either overall (r's = -0.01-0.25, p's > .065), or within each group separately (r's = -0.03-0.43, p's > .068). The number of intrusions here did significantly and positively correlated with TEQ scores in all load (except verbal) conditions overall ($n = 57$, r's = 0.31-0.32, p's < .018), but not within each group separately (r's = -0.22 to 0.44, p's > .086), and with SUIS scores in the no load condition and for the overall sample ($n = 57$, r = 0.31-0.32, p's < .018). Adding TEQ as a covariate to all relevant analyses did not change the pattern of results.

We further explored whether there were group differences in overall ratings of intrusion vividness, distress and newness (Table S4). Because some load conditions had zero intrusions, ratings were not given (becoming 'missing' data). Therefore, these repeated measures with missing data were analysed with mixed effects models (between-group: reminder-plus-Tetris and reminder-only; within-group: no load, visuospatial WM load or verbal WM load at retrieval) which allows the use of all available data without imputing missing values (Field, 2005). Degrees of freedom were rounded up.

[Insert Table S4]

For *vividness* ratings, the main effect of group was not significant, $F < 1$. The main effect of retrieval load was significant, $F(1, 31) = 7.39$, $p = .002$, with post-hoc comparisons revealing that intrusion *vividness* was estimated as significantly lower during both high loads, that is, visuospatial ($M = 27.17$, $SE = 4.37$, $p = .001$) or verbal ($M = 32.44$, $SE = 4.68$, $p = .036$), compared to no load ($M = 41.29$, $SE = 4.79$). No significant differences were observed between visuospatial vs. verbal load ($p = .124$). The group × load interaction was not significant, $F < 1$.

For *distress* ratings. The main effect of group was not significant, $F < 1$. The main effect of retrieval load was significant, $F(1,31) = 4.44$, $p = .020$, with post-hoc comparisons revealing that intrusion *distress* was estimated as significantly lower during visuospatial load only ($M = 21.72$, $SE = 4.45$, $p = .008$) compared to no load ($M = 31.98$, $SE = 4.88$). Distress ratings for verbal load ($M = 27.44$, $SE = 4.84$) lied intermediate (vs. no load, $p = .272$; vs. visuospatial load, $p = .096$). The group × load interaction was not significant, $F(1, 31) = 2.01$, $p = .147$.

Finally, for *nowness* ratings, the main effect of group was not significant, $F < 1$. The main effect of retrieval load was significant, $F(1, 31) = 3.90$, $p = .031$, with post-hoc comparisons revealing that intrusion *nowness* was estimated as significantly lower during both high loads, that is, visuospatial ($M = 21.61$, $SE = 4.19$, $p = .019$) or verbal ($M = 22.37$, $SE = 4.13$, $p = .019$), compared to no load ($M = 33.28$, $SE = 4.85$). No significant differences were observed between visuospatial versus verbal retrieval load ($p = .824$). The group × load interaction was not significant, $F < 1$.

References for the Supplemental Materials

Beck, A. T., Steer, R. A., & Brown, G. K. (1996). *Manual for the Beck Depression Inventory-II*. San Antonio, TX: Psychological Corporation.

Beck, A. T., Steer, R. A., & Garbin, M. G. (1988). Psychometric properties of the Beck Depression Inventory: twenty-five years of evaluation. *Clinical Psychology Review*, *8*(1), 77–10.

Bourne, C., Mackay, C. E., & Holmes, E. A. (2013). The neural basis of flashback formation: the impact of viewing trauma. *Psychological Medicine*, *43*(7), 1521–1532. https://doi.org/10.1017/S0033291712002358

Bremner, M. (1988). *Enquire within upon everything: the complete home reference book.* (1st ed.). Guild Publishing.

Clark, I. A., Holmes, E. A., Woolrich, M. W., & Mackay, C. E. (2016). Intrusive memories to traumatic footage: the neural basis of their encoding and involuntary recall. *Psychological Medicine*, *46*(3), 505–518. https://doi.org/10.1017/S0033291715002007

Deeprose, C., Zhang, S., Dejong, H., Dalgleish, T., & Holmes, E. A. (2012). Imagery in the aftermath of viewing a traumatic film: using cognitive tasks to modulate the development of involuntary memory. *Journal of Behavior Therapy and Experimental Psychiatry*, *43*(2), 758–764. https://doi.org/10.1016/j.jbtep.2011.10.008

Field, A. P. (2005). *Discovering statistics using SPSS (and sex, drugs and rock "n" roll)*. Sage Publications.

Foa, E. B. (1995). *The Posttraumatic Diagnostic Scale manual*. Minneapolis, MN: National Computer Systems.

Gönen, M., Johnson, W. O., Lu, Y., & Westfall, P. H. (2005). The bayesian two-sample t-test. *The American Statistician*, *59*(3), 252–257. https://doi.org/10.1198/000313005X55233

Grey, N., & Holmes, E. A. (2008). "Hotspots" in trauma memories in the treatment of post-traumatic stress disorder: a replication. *Memory*, *16*(7), 788–796. https://doi.org/10.1080/09658210802266446

Holmes, E. A., Grey, N., & Young, K. (2005). Intrusive images and "hotspots" of trauma memories in posttraumatic stress disorder: an exploratory investigation of emotions and cognitive themes. *Journal of Behavioral Therapy and Experimental Psychiatry*, *36*(1), 3–17. https://doi.org/10.1016/j.jbtep.2004.11.002

Holmes, E. A., James, E. L., Coode-Bate, T., & Deeprose, C. (2009). Can playing the computer game "Tetris" reduce the build-up of flashbacks for trauma? A proposal from cognitive science. *PloS One*, *4*(1), e4153. https://doi.org/10.1371/journal.pone.0004153

Holmes, E. A., James, E. L., Kilford, E. J., & Deeprose, C. (2010). Key steps in developing a cognitive vaccine against traumatic flashbacks: visuospatial Tetris versus verbal Pub Quiz. *PloS One*, *5*(11), e13706. https://doi.org/10.1371/journal.pone.0013706

James, E. L., Bonsall, M. B., Hoppitt, L., Tunbridge, E. M., Geddes, J. R., Milton, A. L., & Holmes, E. A. (2015). Computer game play reduces intrusive memories of experimental trauma via reconsolidation-update mechanisms. *Psychological Science*, *26*(8), 1201–1215. https://doi.org/10.1177/0956797615583071

James, E. L., Lau-Zhu, A., Clark, I. A., Visser, R. M., Hagenaars, M. A., & Holmes, E. A. (2016). The trauma film paradigm as an experimental psychopathology model of psychological trauma: intrusive memories and beyond. *Clinical Psychology Review*, *47*,

106–142. https://doi.org/10.1016/j.cpr.2016.04.010

James, E. L., Lau-Zhu, A., Tickle, H., Horsch, A., & Holmes, E. A. (2016). Playing the computer game Tetris prior to viewing traumatic film material and subsequent intrusive memories: examining proactive interference. *Journal of Behavior Therapy and Experimental Psychiatry*, *53*, 25–33. https://doi.org/10.1016/j.jbtep.2015.11.004

Levine, B., Svoboda, E., Hay, J. F., Winocur, G., & Moscovitch, M. (2002). Aging and autobiographical memory: dissociating episodic from semantic retrieval. *Psychology and Aging*, *17*(4), 677–689. https://doi.org/10.1037//0882-7974.17.4.677

Mace, J. (2004). Involuntary autobiographical memories are highly dependent on abstract cuing: the Proustian view is incorrect. *Applied Cognitive Psychology*, *18*(7), 893–899. https://doi.org/10.1002/acp.1020

McKinnon, M. C., Palombo, D. J., Nazarov, A., Kumar, N., Khuu, W., & Levine, B. (2014). Threat of death and autobiographical memory: a study of passengers from flight AT236. *Clinical Psychological Science*, *3*(4), 487–502. https://doi.org/10.1177/2167702614542280

Rajaram, S. (1993). Remembering and knowing: two means of access to the personal past. *Memory and Cognition*, *21*(1), 89–102.

Reisberg, D., Pearson, D. G., & Kosslyn, S. M. (2003). Intuitions and introspections about imagery: the role of imagery experience in shaping an investigator's theoretical views. *Applied Cognitive Psychology*, *17*(2), 147–160. https://doi.org/10.1002/acp.858

Schneider, W., Eschman, A., & Zuccolotto, A. (2002). *E-Prime User's Guide.* Pittsburgh: Psychology Software Tools Inc.

Spielberger, C. D., Gorsuch, R. L., Lushene, R., Vagg, P. R., & Jacobs, G. A. (1983). *Manual for the State-Trait Anxiety Inventory*. Palo Alto, CA: Consulting Psychologist Press.

Spielberger, C. D., Reheiser, E. C., Owen, A. E., & Sydeman, S. J. (2004). Measuring the psychological vital signs of anxiety, anger, depression, and curiosity in treatment planning and outcomes assessment. In *The Use of Psychological Testing for Treatment Planning and Outcomes Assessment: Volume 3: Instruments for Adults (3rd ed)* (pp. 421–447).

Yonelinas, A. P. (2002). The nature of recollection and familiarity: a review of 30 years of research. *Journal of Memory and Language*, *46*(3), 441–517. https://doi.org/10.1006/JMLA.2002.2864

Table S1

Means and Standard Deviations by Group for Baseline Measures, Mood Ratings and Task Manipulation Checks in Experiment 1

	Reminder-plus-Tetris (n = 23)		Reminder-only (n = 23)	
	n		*n*	
Gender (females)	15		13	
	M	*SD*	*M*	*SD*
Age	27.39	6.87	27.91	7.17
BDI-II	6.61	6.51	8.26	6.40
STAI-T	37.87	8.69	38.96	9.83
TEQ	1.61	1.73	1.48	1.38
SUIS	39.74	5.37	39.26	8.98
Pre-film negative mood	1.38	1.91	2.72	3.93
Post-film negative mood	9.32	7.88	8.96	7.04
Film attention	9.26	1.21	9.43	0.73
Personal relevance of film	3.91	3.25	4.35	2.89
Demand ratings	-1.26	3.70	-1.83	3.30
Diary compliance	8.35	1.97	8.39	1.08

Note. BDI-II = Beck Depression Inventory; STAI-T = State Trait Anxiety Inventory – Trait Version; TEQ = Traumatic Experience Questionnaire; SUIS = Spontaneous Use of Imagery Scale.

Table S2

Means and Standard Deviations by Group for Baseline Measures, Mood Ratings and Task Manipulation Checks in Experiment 2

	Reminder-plus-Tetris (n = 18)		Reminder-only (n = 18)	
	n		*n*	
Gender (females)	10		9	
	M	*SD*	*M*	*SD*
Age	26.39	7.24	24.94	7.00
BDI-II	5.50	3.47	6.72	4.60
STAI-T	35.11	9.92	39.22	8.23
TEQ	1.39	1.75	1.00	1.09
SUIS	39.56	5.58	40.06	6.26
Pre-film negative mood	1.84	1.32	4.07	3.95
Post-film negative mood	7.66	4.85	10.42	6.54
Attention to film	9.17	0.71	9.22	1.00
Film relevance	3.44	2.46	3.83	2.90
Demand ratings	- 2.94	4.52	-1.83	3.47
Diary compliance	8.94	1.00	8.06	1.16

Note. BDI-II = Beck Depression Inventory; STAI-T = State Trait Anxiety Inventory – Trait Version; TEQ = Traumatic Experience Questionnaire; SUIS = Spontaneous Use of Imagery Scale.

Table S3

Means and Standard Deviations by Group for Baseline Measures, Mood Ratings and Task Manipulation Checks in Experiment 3

	Reminder-plus-Tetris (*n* = 19)		Reminder-only (*n* = 19)		Tetris-only (*n* = 19)	
	n		*n*		*n*	
Gender (females)	11		13		10	
	M	*SD*	*M*	*SD*	*M*	*SD*
Age	27.26	7.81	25.32	5.40	28.05	6.90
BDI-II	4.95	5.34	4.68	4.67	5.37	5.77
STAI-T	36.32	8.35	33.53	8.07	35.89	10.55
TEQ	0.58	0.77	1.32	1.20	0.74	1015
SUIS	35.84	9.91	36.68	8.89	39.37	7.83
Pre-film negative mood	1.87	3.21	1.54	2.37	2.11	2.49
Post-film negative mood	8.57	7.26	8.32	6.20	7.55	5.28
Film attention	9.53	0.96	9.53	0.61	9.37	0.76
Film relevance	3.00	2.40	4.37	2.89	3.68	2.47
Demand ratings	-1.32	2.96	-1.42	3.19	-1.65	3.62

Note: BDI-II = Beck Depression Inventory; STAI-T = State Trait Anxiety Inventory – Trait Version; TEQ = Traumatic Experience Questionnaire; SUIS = Spontaneous Use of Imagery Scale.

Table S4

Means and Standard Deviations by Group (Reminder-plus-Tetris vs. Reminder-only) for Ratings of Intrusion Vividness, Distress and Nowness in Experiment 3

	Reminder-plus-Tetris			Reminder-only		
	n	*M*	*SD*	*n*	*M*	*SD*
Vividness						
No load	15	42.52	25.36	18	40.69	29.90
Visuospatial load	12	27.28	21.83	19	27.58	26.86
Verbal load	11	31.61	18.10	16	37.63	29.41
Distress						
No load	15	32.21	22.78	18	31.69	33.47
Visuospatial load	12	22.01	17.97	19	23.25	29.19
Verbal load	11	23.88	17.00	16	37.58	30.35
Nowness						
No load	15	33.54	29.09	18	33.07	29.41
Visuospatial load	12	27.43	20.16	19	16.83	21.97
Verbal load	11	25.62	22.57	16	23.25	24.39
